# Changes of RAS Pathway Phosphorylation in Lymphoblastoid Cell Lines from Noonan Syndrome Patients Carrying Hypomorphic Variants in Two NS Genes

**DOI:** 10.3390/ijms24044035

**Published:** 2023-02-17

**Authors:** Viviana Tritto, Daniele Capitanio, Cecilia Gelfi, Paola Riva

**Affiliations:** 1 Dipartimento di Biotecnologie Mediche e Medicina Traslazionale, Università degli Studi di Milano, 20054 Segrate, Italy; 2 Dipartimento di Scienze Biomediche per la Salute, Università degli Studi di Milano, 20133 Milan, Italy; 3 IRCCS Istituto Ortopedico Galeazzi, 20161 Milan, Italy

**Keywords:** Noonan syndrome, RAS pathway genes, hypomorphic variants, additive effect, phosphoproteome, proteome

## Abstract

Noonan syndrome (NS) is an autosomal dominant multisystem disorder, characterized by variable expressivity and locus heterogeneity, being caused by mutations in one of a subset of RAS pathway genes. Nevertheless, for 20–30% of patients it is not possible to provide molecular diagnosis, suggesting that further unknown genes or mechanisms are involved in NS pathogenesis. Recently, we proposed a digenic inheritance of subclinical variants as an alternative NS pathogenic model in two NS patients negative for molecular diagnosis. They showed hypomorphic variants of RAS pathway genes co-inherited from both their healthy parents that we hypothesized to generate an additive effect. Here, we report on the phosphoproteome and proteome analysis by liquid chromatography tandem mass spectrometry (LC-MS/MS) performed on the immortalized peripheral blood mononuclear cells (PBMCs) from the two above trios. Our results indicate that the two unrelated patients show overlapped profiles in both protein abundances and their phosphorylation levels not reached by their parents. IPA software predicted RAS-related pathways as significantly activated in the two patients. Interestingly, they remained unchanged or only slightly activated in both patients’ parents. These findings suggest that the presence of one subclinical variant can activate the RAS pathway below the pathological threshold, which can instead be exceeded by the additive effect due to the co-presence of two subclinical variants causing NS, supporting our digenic inheritance hypothesis.

## 1. Introduction

Noonan syndrome (NS) is an autosomal-dominant disorder with a prevalence of 1:1000–2500 and is included in the group of RASopathies, being caused by RAS/MAPK (mitogen-activated protein kinase) signal transduction pathway deregulation. Common clinical signs are hypertelorism, downslanting of the palpebral fissures, ptosis, low-set, posteriorly rotated ears, and webbed or short neck [1,2,3,4]. More than 80% of NS patients exhibit congenital heart diseases, pulmonary valve stenosis, and hypertrophic cardiomyopathy [5,6]. Other clinical manifestations are cryptorchidism, bleeding disorders, mild neurocognitive delay and pectus deformity, and an increased risk of developing myeloproliferative disorders [3,7,8]. Due to the considerably variable expressivity, some NS adults are diagnosed after the birth of an affected child [9,10,11].

Pathogenic variants involve different genes: *PTPN11* (in 40–50% of the patients), *SOS1* (10–20%), *RAF1* (3–17%), *RIT1* (9%), and a group of genes *KRAS*, *NRAS*, *BRAF*, *SHOC2*, *MAP2K1*, *CBL*, *LZTR1*, *SOS2*, *RRAS*, *A2ML1*, and *CDC42*, accounting for 1–5% of NS patients [12]. Furthermore, a few NS and NS–like cases have been reported to show copy number variations (CNV) encompassing NS-associated genes [13,14]. Nevertheless, NS pathogenesis is unknown for 20–30% of patients and locus heterogeneity is not sufficient to explain the variable expressivity, pinpointing the need for identifying new genes or mechanisms responsible for NS pathogenesis [7,15].

Interestingly, we reported on a NS patient with a severe phenotype who showed multiple NS clinical features, including facial dysmorphisms, pulmonary valve stenosis, hypertrophic cardiomyopathy, mitral valve prolapse, cutaneous xerosis and keratosis pilaris, a café-au-lait spot on the abdomen, hypertrichosis, left foot fifth finger overlapping the fourth finger, a mild thorax deformity, and who carried a de novo *RAF1* variant and an *SOS1* variant inherited from his father and grandfather, both of whom presented a subclinical NS phenotype [10]. An additive effect on RAS pathway activation of the subclinical/hypomorphic *SOS1* variant has been hypothesized, thus contributing to the severity of the disease in the patient. More recently, in two out of eight NS patients negative for molecular NS diagnosis and for the presence of CNVs, we described co-inheritance of a hypomorphic variant from both their healthy parents: c.650C>T *LRP1* and c.1964C>T *SOS1* in one patient, and c.136C>T *LRP1* and c.355T>C/c.1430C>T *LZTR1* in the other, prompting us to hypothesize their additive effect in the patients, leading to the RAS pathway hyperactivation threshold causing the disease [16]. Our evidence also suggests that the modulation of RAS pathway activity, through the additive effect of hypomorphic variants, could have a role in the variable expressivity of NS. The digenic inheritance of hypomorphic mutations in NS would introduce a new paradigm in the pathogenesis of the disease, as well as in the pipeline design, where variants that would be excluded on the basis of their presence in the average population, even if singularly and with a low frequency, should be considered.

To investigate this hypothesis, we analyzed proteome and phosphoproteome of the two described trios [16] to verify if phosphorylation levels, in particular that of RAS/RAS-related pathway effectors, were altered not only in patients but also in healthy parents carrying one of the two hypomorphic variants, with a threshold too low for NS clinical manifestation.

Phosphoproteome analysis should help to detect biologically relevant phosphorylation key events involved in specific pathways [17]. Nevertheless, at the time of writing only a few studies aimed at analyzing the dysregulation of phosphoproteins and their biological implications in RASopathies have been carried out. Moreover, a comprehensive landscape of protein–protein interactions concerning all RAS and correlated effectors is still lacking.

Proteomic and phosphoproteomic analyses allow the identification of changes in the expression, composition, modification status, and phosphorylation level of proteins at a specific time, in specific tissues, and under specific conditions to unravel biological phenomena such as signal transduction, metabolic pathways, and cell differentiation. This possibly addresses the identification of key factors involved in functional regulations revealing disease target proteins and putative pharmacological targets.

Studies carried out on NF1-null cells derived from malignant peripheral nerve sheath tumors (MPNST) showing RAS cascade hyperactivation were characterized by a deep phosphoproteome remodeling [18], and the phosphoproteome was assessed by high throughput mass spectrometry also in NS and NS with multiple lentigines (NSML) [19,20,21].

In the present study, the quantitative proteome/phosphoproteome carried out in peripheral blood mononuclear cells (PBMCs) from NS patients identified a specific phosphoproteomic pattern that could be used as a predictive molecular NS signature in asymptomatic parents, associated with a reproductive risk. The study provided evidence of the role of phosphosignaling in RAS pathway related proteins both in healthy parents carrying a subclinical RAS pathway variant and in a NS affected child carrying two co-inherited subclinical variants.

## 2. Results

### 2.1. Phosphoproteome and Proteome Analysis by LC-MS/MS

To investigate possible changes in the phosphoproteome of patients, characterized by two mutated genes, and their parents, characterized by one mutated gene in the RAS pathway, phosphoproteome and proteome analysis by liquid chromatography tandem mass spectrometry (LC-MS/MS) on the immortalized PBMCs was performed.

#### 2.1.1. Phosphorylated Protein Levels Changes

After phosphoproteome enrichment, samples underwent trypsin digestion and LC-MS/MS analysis. Statistically significant differences were determined by comparing each sample with age-matched controls (young and adult, respectively, for affected children and parents) (Table S1). 

Family 53. The patient showed 153 proteins with increased abundances and 163 with decreased abundances out of the 894 identified phosphoproteins. 

The father showed 135 and 263 proteins with increased and decreased abundances, respectively, out of 868 detected phosphoproteins. The mother showed 129 proteins with increased abundances and 314 decreased out of the 859 phosphoproteins identified. 

Family 67. The patient showed 175 and 176 proteins increased and decreased in abundance, respectively, out of 891 phosphoproteins identified.

The father showed 130 and 140 proteins with abundance levels increased and decreased, respectively, out of 884 phosphoproteins detected. The mother showed 156 and 232 proteins with increased and decreased abundances, respectively, out of 860 phosphoproteins. 

In the two unrelated patients, the phosphoproteome analysis revealed the same number of decreased and increased phosphoproteins, while in three out of four parents the phosphoproteins with increased levels were half of those showing reduced levels (Table S1).

#### 2.1.2. Evaluation of the Proteome Phosphorylation Pattern

Given alterations found in the phosphoprotein pattern of NS patients, to understand whether the change in the phosphoproteome was due to alterations in total protein or a rise in kinase activity, the phosphoproteins’ abundance, obtained from the phosphoproteome analysis, were normalized in respect to their proteome abundance assessed for each patient and parent vs. healthy controls (Table S2). An increased phosphorylation level, or hyper-phosphorylation, was defined as the condition in which an increase in phosphorylated protein abundance and an unchanged or decreased level of total protein were found. The ratio between the number of phosphoproteins and the number of proteins identified remained constant in the different samples. The proteins present in both datasets ranged from 37.0% to 41.5% of the phosphoproteome and 45.5% to 50.0% of the proteome (Figure S1). 

In young and adult healthy control groups, 367 and 350 phosphoproteins were identified, respectively, both in proteome and phosphoproteome, in common between the two samples belonging to each group. The comparison between the values obtained from the phosphorylation analysis of young healthy controls and those of adult controls showed 50% differentially phosphorylated proteins, three-fifths of which were hyper-phosphorylated in the young controls (Table S2). In family 53, the phosphorylation level in the patient was increased in 106 proteins and decreased in 44, out of 270 phosphorylated proteins. In the father, among the 254 proteins detected both in proteome and in phosphoproteome, 49 and 57 phosphoproteins showed an increase and decrease in phosphorylation levels, respectively. Looking at the 256 phosphoproteins detected in the mother, 43 appeared hyper-phosphorylated and 73 hypo-phosphorylated. In Family 67, 343 phosphoproteins were identified both in the proteome and phosphoproteome of the patient, of which 114 showed increased and 55 showed decreased phosphorylation levels. The father showed 71 hyper-phosphorylated and 68 hypo-phosphorylated phosphoproteins out of the 335 proteins analyzed. In the mother, 71 and 73 phosphoproteins were increased and decreased in phosphorylation levels, respectively, among the 338 analyzed phosphoproteins. 

Interestingly, in the two patients, more than two-thirds of proteins with altered phosphorylation levels displayed an increased phosphorylation amount. Instead, three out of four parents showed a similar percentage of proteins with greater and lesser phosphorylation levels compared with controls (Table S2).

Of note considering the quantitative protein expression (Table S3) and changes in the degree of phosphorylation (Table S2), the two patients showed a statistically significant increase in the percentage of hypo-expressed but hyper-phosphorylated proteins and a decrease in overexpressed but hypo-phosphorylated proteins, compared with their parents, with a *p*-value < 0.05 (Table S4).

### 2.2. Comparison of Proteins Exhibiting Changes in Phosphorylation Level

**Figure 1:**
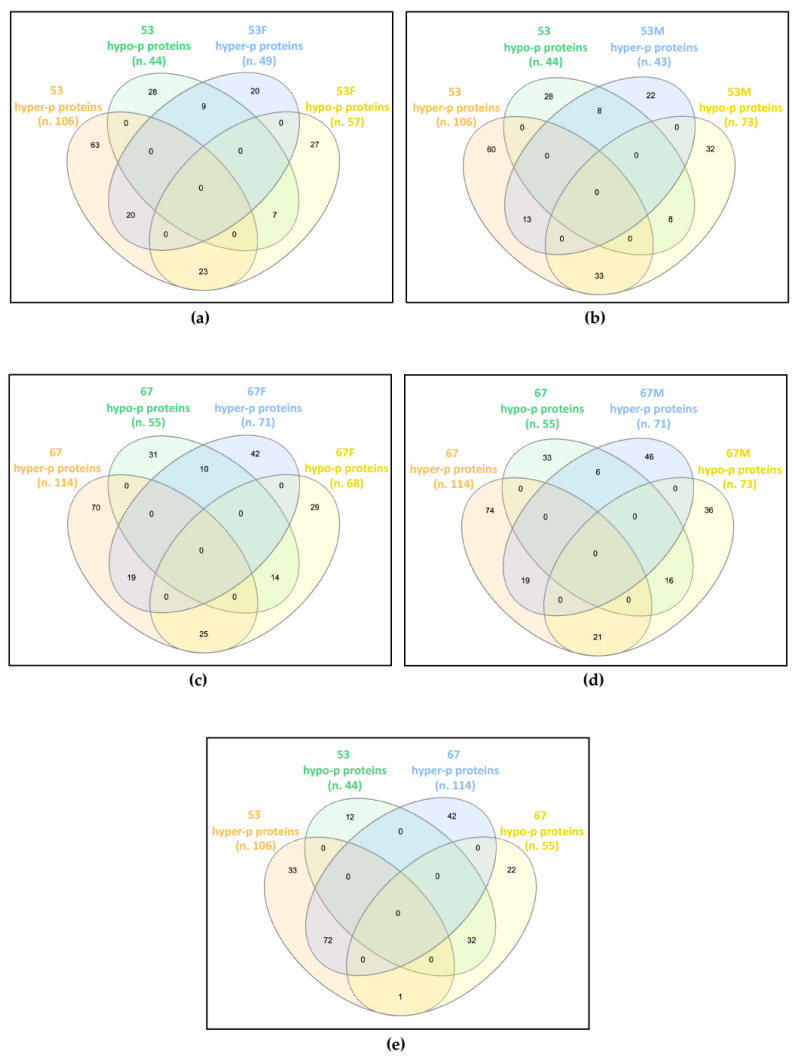
Common differentially phosphorylated proteins in: (**a**) patient 53 (53) and his father (53F); (**b**) patient 53 and his mother (53M); (**c**) patient 67 (67) and her father (67F); (**d**) patient 67 and her mother (67M); (**e**) patients 53 and 67. The Venn diagrams, drawn using InteractiVenn tool, show that the two patients widely share the altered phosphorylation profile with each other, and only partially with their own parents. n., number of differentially phosphorylated proteins (p proteins).

The datasets of proteins with altered phosphorylation degrees, obtained by comparing each analyzed sample against its respective control (Table S2), were compared and any common trends in protein phosphorylation profiles were highlighted (Figure 1).

In family 53, the patient shared 59 proteins showing deregulated phosphorylation with his father, of which 20 were hyper-phosphorylated and seven were hypo-phosphorylated in both, while 23 were hyper-phosphorylated in the patient and hypo-phosphorylated in the father, and nine were hypo-phosphorylated in the patient and hyper-phosphorylated in the father (Figure 1a). The patient and his mother showed 62 proteins with dysregulated phosphorylation in common, of which 13 and eight were increased and decreased in phosphorylation levels, respectively, while 33 and eight showed hyper- and hypo-phosphorylation levels in the patient and an opposite trend in the mother (Figure 1b).

In family 67, the patient shared 68 and 62 proteins with altered phosphorylation levels compared with her father and mother, respectively. Among proteins shared by the patient and the father, 19 were hyper-phosphorylated and 14 were hypo-phosphorylated in both, 25 were hyper-phosphorylated in the patient and hypo-phosphorylated in the father, while for 10 proteins the reverse trend was observed (Figure 1c). Among proteins shared by the patient and the mother, 19 hyper- and 16 hypo-phosphorylated proteins were detected in both, while 21 hyper- and six hypo-phosphorylated were found in the patient, with a reverse trend in the mother (Figure 1d). 

By comparing the differentially phosphorylated proteins of the two unrelated patients, 104 proteins with the same trend were identified, 72 hyper- and 32 hypo-phosphorylated, and a single protein showed an opposite trend, i.e., hyper-phosphorylated in patient 53 and hypo-phosphorylated in patient 67 (Figure 1e).

Interestingly, while each patient only partially shared the impaired phosphorylation profile identified in their parents, the two patients have widely overlapped profiles, presenting about two-thirds of their differentially phosphorylated proteins in common and with the same trend towards hyper- or hypo-phosphorylation.

### 2.3. Bioinformatics Analysis of Proteomics Results

Results from label-free proteomics analysis have been analyzed utilizing Ingenuity Pathway Analysis (IPA) software to predict biochemical pathways associated with differentially expressed proteins. In particular, datasets of changed phosphoproteins/total proteins were analyzed. In the first step, a core analysis, starting from the list of changed proteins with their *p*-values and fold changes, was performed. This analysis allowed researchers to identify canonical pathways predicted to be involved in response to phosphoproteome changes.

Seven pathways significantly changed (Fisher’s right-tailed exact test *p*-value < 0.05 and z-score ≥ 2 or ≤−2) were identified. As reported in Table 1, in patients 53 and 67 vs. young healthy controls, the main activated pathways highlighted by IPA software were RHOA signaling, CD28 signaling in T helper cells, signaling by Rho Family GTPases, remodeling of epithelial adherens junctions, fMLP signaling in neutrophils, CDC42 signaling, and the synaptogenesis signaling pathway. In both patient’s parents (53F-53M, 67F-67M) vs. age-matched controls, the same pathways were unchanged or predicted as only slightly activated (z-score < |2|). The pathways that emerged from the IPA enrichment analysis were in network with the RAS pathway. The pathways’ branches connected to RAS signaling are circled in red in Figure S2a–g.

### 2.4. Changes in the Phosphorylation Cascade of the RAS Signaling Pathway

To verify whether the identified proteome phosphorylation profile could be consistent with a Noonan-like phenotype shown by the two patients, their differentially phosphorylated and/or abundant phosphoproteins were compared with a list of RAS pathway proteins (Table S5). Furthermore, the same analysis was performed on their parents to understand if the presence of the single hypomorphic variant, previously identified by genetic screening in these subjects and transmitted by each of them to their respective child [16], could have determined alterations below the clinical threshold of RAS pathway proteins.

**Figure 2:**
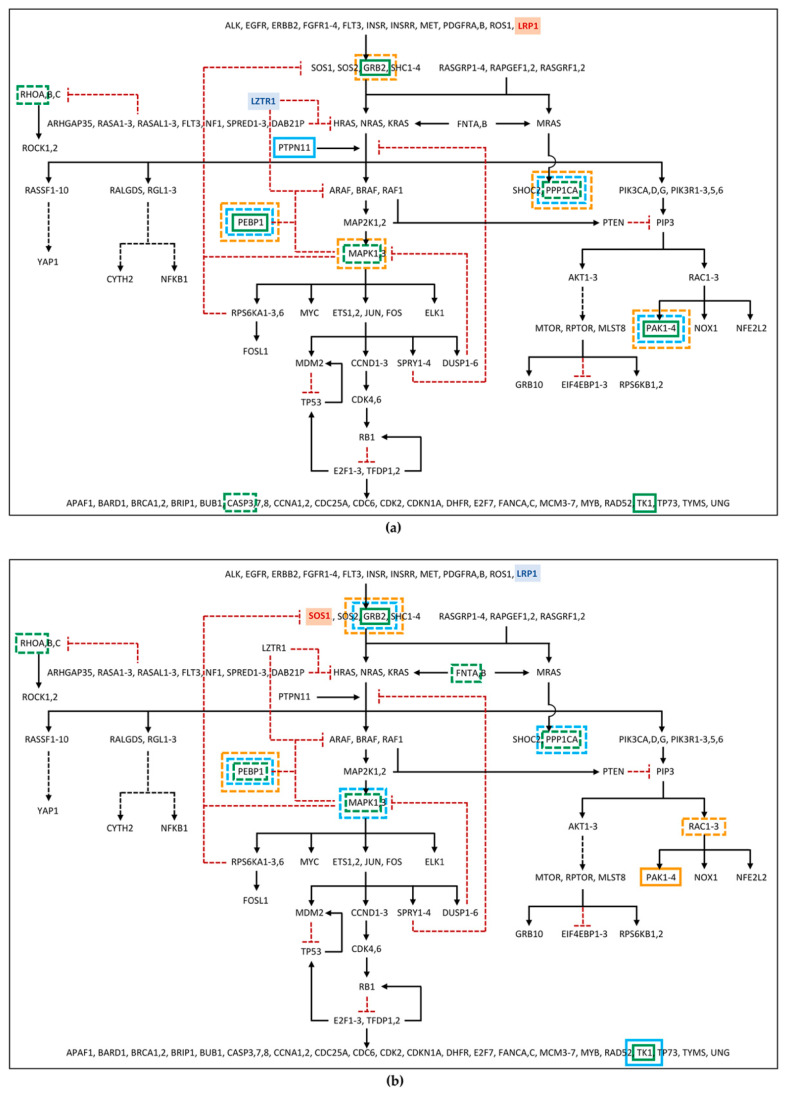
RAS pathway diagram showing changes in the phosphorylation cascade of: (**a**) Family 53; (**b**) Family 67. The diagram shows proteins, represented by gene symbol, belonging to RAS and related pathways, from the receptors and messengers, at the top of the diagram, up to the RAS proteins, which in turn signal through five families of alternative effectors, acting on many downstream targets. Black arrows represent activation signals, while dotted red “T” lines indicate inhibitory signals. Dashed black arrows indicate that the signaling pathway intermediaries are not shown. The gene with the hypomorphic variant in father and proband is shown in blue, the one mutated in mother and proband in red. Phosphoproteins showing impaired phosphorylation or abundance are enclosed in a box (in green for patient, in light blue for father, in orange for mother), with continuous or dotted line if we have detected high or low levels, respectively. For phosphoprotein fold changes, see Supplementary Data available on Dataverse (https://doi.org/10.13130/RD_UNIMI/PKZ7FJ, deposited on 30 October 2022). The diagram was adapted from RAS Pathway v2.0, originally published by the National Cancer Institute (https://www.cancer.gov/research/key-initiatives/ras/ras-central/blog/2015/ras-pathway-v2, accessed on 1 June 2022).

Among the differentially phosphorylated proteins belonging to the RAS pathway, in family 53, PEBP1 and GRB2 were identified as hyper-phosphorylated in the patient, but hypo-phosphorylated in the mother. Furthermore, RHOA was found hypo-phosphorylated in patient 53 (Figure 2a; Table S6). In family 67, the GRB2 hyper-phosphorylation and the hypo-phosphorylation of MAPK1 and RHOA were found in the patient, while hypo-phosphorylation of MAPK1 and GRB2, in the father, and of PEBP1, in the mother, was detected (Figure 2b; Table S6). Furthermore, proteins belonging to the RAS pathway were also searched among those showing an unchanged degree of phosphorylation but an altered expression level in the proteome, as deregulation in protein expression could affect the amount of signal transmitted by the phosphorylation cascade. In this group, PEBP1 was found down-regulated in father 53, patient 67, and father 67, and GRB2 was found down-regulated only in mother 67 (Figure 2; Table S7). At last, RAS pathway proteins were identified among the phosphoproteins for which it was not possible to determine the phosphorylation level variation, as the protein itself was not detected in the proteome assay, but which showed an altered abundance in the phosphoproteome profile. Among these phosphoproteins, PPP1CA, MAPK1, and CASP3 in patient 53; PPP1CA and PAK2 in father 53; PPP1CA, MAPK1, and PAK2 in mother 53; FNTA and PPP1CA in patient 67; PPP1CA in father 67; and RAC2 in mother 67 were all found to be reduced in the abundance level, while TK1 and PAK2 in patient 53, PTPN11 in father 53, TK1 in patient 67 and in father 67, and PAK1 in mother 67 showed a rise in the abundance level (Figure 2; Table S8).

As expected, more phosphoproteins with impaired levels of phosphorylation/abundance were identified in the two unrelated patients compared with their parents. Of note, patients showed a comparable dysregulation profile of the phosphorylation cascade, which was only partially shared by their parents.

### 2.5. Validation by Immunoblotting

**Figure 3:**
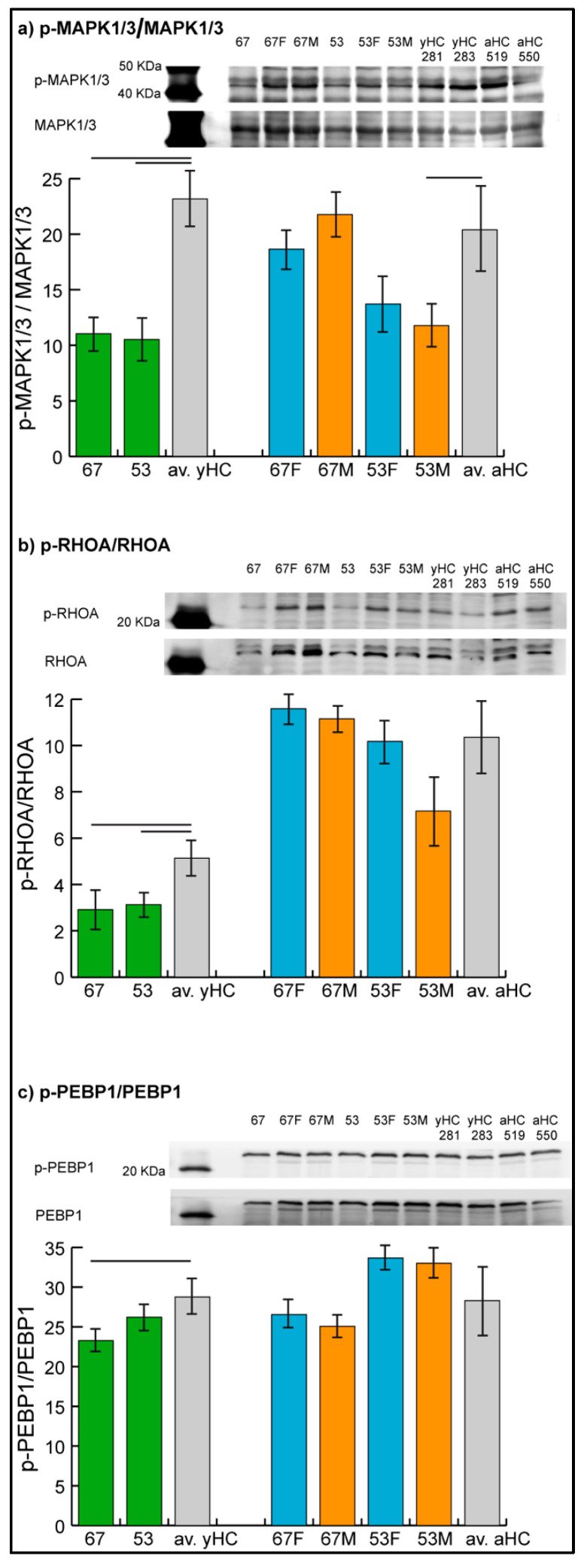
Representative immunoblot images of phosphorylated mitogen-activated protein kinase 1/3, MAPK1/3 (**a**), Ras homolog family member A, RHOA (**b**), and phosphatidylethanolamine binding protein 1, PEBP1 (**c**). Histograms show the ratio of phosphorylated over the total protein band intensity (mean ± SD) in patients 53 and 67, in their parents (53F, 53M, 67F, 67M) and in two unrelated young (yHC 281 and 283) and adult (aHC 519 and 550) controls. Significant differences between each sample and the means of the two respective controls (averaged yHC and aHC) were computed applying the *t*-test (n = 2, significance threshold: *p*-value < 0.05) and were indicated by the horizontal lines.

The levels of phosphorylated MAPK1/3, RHOA, and PEBP1 were assessed by immunoblotting (Figure 3), while the phosphorylation level of GRB2 could not be evaluated due to technical problems given by nonspecificity of the related antibody in the assay. 

Results confirmed the hypo-phosphorylation of MAPK1/3 and RHOA in both patients and the hypo-phosphorylation of PEBP1 in patient 67. Hyper-phosphorylation of PEBP1 was not confirmed in patient 53 by immunoblot analysis, in which the phosphorylation level of PEBP1 was found to be not significantly changed in the patient compared with controls.

In parents, unchanged levels of the three proteins were observed, with the exception of MAPK1/3, which showed a trend of hypo-phosphorylation (*t*-test, n = 2, *p*-value < 0.05) in mother 53.

## 3. Discussion

In 20–30% of patients with NS, the genetic cause of the disease is not identified. Therefore, we hypothesized an alternative hereditary mechanism to monogenic inheritance. We previously described hypomorphic variants in more than one RAS pathway gene co-present in two NS sporadic patients, negative after conventional mutation analysis, leading to us speculate an additive effect of the two hypomorphic variants on RAS pathway activation and a new NS genetic mechanism based on digenic inheritance [16]. In fact, both patients had healthy parents, all carrying one of the variants present in their child that presumably generated a perturbation of the RAS pathway not sufficient to cause a NS phenotype, differently from their child who, for the presence of variants in two genes, showed a threshold of phosphorylation that caused the RASopathy. According to this hypothesis, we expected a subthreshold activation of the RAS pathway in healthy parents that could be detected by means quantitative analysis of proteome and phosphoproteome, where the biomarker was the level of phosphorylated proteins of RAS and related pathways. We expect healthy parents to have a higher level of phosphorylated proteins than controls but less high than those of their NS-affected children. Here, we investigated the proteome and phosphoproteome by LC-MS/MS analyzing in the two trios both phosphorylation level and protein expression level to distinguish whether any alteration in phosphorylation degree was due to increased phosphorylation, increased abundance of phosphorylated proteins, or both mechanisms. 

We here provide evidence on the different modulation of protein phosphorylation in healthy parents and their affected child. The phosphorylation grade, obtained by the ratio between the abundance (LFQ intensity) of the specific phosphorylated protein and the total amount of the protein itself, resulted differently in parents and their child in relation to controls. This analysis indicated that the phosphorylation level of the single proteins analyzed varied in parents compared with controls. Interestingly, most of the variation in patients consisted of increased protein phosphorylation compared with their parents and controls. The patients showed a similar pattern of phosphorylation among themselves, and different patterns compared with their healthy parents: this observation indicates that there is a correlation between a specific phosphorylation pattern and disease manifestation. The presence of increased phosphorylation and the absence of a specific phosphorylated protein pattern in the parents was expected, as they were healthy. Moreover, the parameter used was suitable to study the modulations of phosphorylation when the RAS pathway was affected.

The RAS/MAPK signaling pathway is a chain of proteins in the cell that communicates a signal from a receptor on the cell surface to the DNA and has a relevant impact on human health, as RAS pathway-activating mutations can result in a group of developmental conditions, named “RASopathies”, and can also contribute significantly to cancer [22]. 

The number of large-scale mass spectrometry-based phosphoproteome studies has swelled over the past decade, initiating its application to RASopathies. However, only a few studies focused on dysregulated phosphoproteins and their biological implications [23,24]. Phosphoproteomics data in RASopathies are still scarce and their potential to decipher crucial signaling pathways involved in this family of disorders needs to be considered. 

Given that RAS generally controls phosphorylation capacity [25,26], this is in line with our findings showing altered phosphorylation patterns in patients with RASopathy. If we consider all proteins with altered phosphorylation and search for the pathways in which these proteins are involved, pathways related to the RAS pathway emerge. Interestingly, variation in activation of the same pathways was detected in affected patients and also in their parents, although with a lower level of activation in the latter, which was not statistically significant. These data are in line with our hypothesis, namely that significant activation of the pathways of interest is shown by patients, while their healthy parents show only a trend of activation, not significant, as expected. In our NS patients, the two hypomorphic variants, each inherited from a healthy parent, may contribute to the pathological threshold of pathway activation and, by an additive effect, cause the disease. This hypothesis is consistent with the finding that, focusing on RAS pathway proteins, phosphoproteins with impaired levels of phosphorylation/abundance were identified both in the two unrelated patients and in their parents, by LC-MS/MS analysis. Changes in the phosphorylation degree of some of these proteins were confirmed by immunoblotting in patients, while it was not possible to confirm variations of milder intensity, such as those found in some of the parents, probably due to the lower sensitivity and greater technical variability of the Western blot technique compared with mass spectrometry approaches [27]. Functional studies need to be carried out to validate the hypothesis suggested by the evidence obtained. 

Interestingly, among the differentially phosphorylated proteins belonging to the RAS pathway, identified by LC-MS/MS analysis, and confirmed by immunoblotting, RHOA was found hypo-phosphorylated in patients 53 and 67, even if IPA software predicted an activation of RHOA signaling in the same patients. Although the two results may seem discordant, it should be noted that, besides hypo-phosphorylation of RHOA, the IPA software considered alterations in the phosphorylation level of additional components of RHOA signaling present in our dataset (Figure S3), on which other pathways may also act. Therefore, the pathway activation consists of the overall outcome of the prediction.

Based on our findings, we think that quantitative proteomic studies provide a comprehensive proteomic signature for PBMCs from patients. In particular, the identification of specific phosphoproteome patterns in NS patients could provide data that direct the identification of druggable target genes, resulting in improved personalized medicine, helping to set up an effective therapy for NS, as well as potentially revealing a predictive molecular NS signature in asymptomatic individuals associated with a reproductive risk. 

## 4. Materials and Methods

### 4.1. Cell Culture

Epstein–Barr Virus (EBV) immortalized PBMCs were provided by Galliera Genetic Bank, from lymphocytes of the two patients, their parents, and four healthy controls, including two children (cell lines 281 and 283) and two adults (cell lines 519 and 550). The immortalized cells were maintained in RPMI 1640 medium without L-Glutamine with phenol red (Euroclone, Milan, Italy) supplemented with 10% fetal bovine serum (FBS South America origin EU Approved, Euroclone, Milan, Italy), 1% L-Glutamine 100X 200 mM (Euroclone, Milan, Italy), and 1% penicillin–streptomycin solution 100X (Euroclone, Milan, Italy) at 37 °C in a 5% CO_2_ atmosphere. Each cell line was synchronized with thymidine treatment, which requires an inoculum of 500,000 cells/mL to be cultured for 24 h in a complete medium enriched with 1% thymidine 200 mM (Merck Life Science, Milan, Italy), capable of blocking cells in the G1 phase, and for the following 24 h in complete medium without thymidine, to restart growth starting from the same stage of the cell cycle, at 37 °C in a 5% CO_2_ atmosphere. At the end of the treatment, the cells were washed with HEPES 50 mM (Merck Life Science, Milan, Italy), to prevent cryo-induced pH changes, and pellets of 5 × 10^6^ cells were stored at −80 °C.

### 4.2. Protein Extraction

For label-free proteomics analysis, each cell pellet (5 × 10^6^ cells) was suspended in 200 µL cell lysis buffer (Pierce, mass spec sample prep kit for cultured cells, Cat.# 84840, Thermo Fisher Scientific, Rodano, Italy) and processed following the protocol. Lysates were incubated at 95 °C for 5 min, sonicated on ice and clarified by centrifugation at 16,000× *g* for 10 min at 4 °C. Protein quantitation with BCA protein assay kit (Pierce, Cat.# 23225, Thermo Fisher Scientific, Rodano, Italy) was then performed, and protein extracts (200 µg) were reduced with DTT (10 mM final concentration) for 45 min at 50 °C.

### 4.3. Phosphoprotein Enrichment

Cells (2 × 10^7^) were washed in a non-phosphate-based buffer (50 mM HEPES, pH 7.0) and cell pellets were processed following the Pierce Phosphoprotein enrichment kit instructions (Pierce, Cat.# 90003, Thermo Fisher Scientific, Rodano, Italy). Following cell lysis, protein extracts were quantitated with BCA protein assay kit, diluted to 0.5 mg/mL in lysis buffer and loaded onto the enrichment columns (2 mg total protein load). Columns were incubated on a rocker for 30 min at 4 °C. Following incubation, resin was rinsed three times in lysis buffer and phosphoproteins eluted with five washes in elution buffer without CHAPS (5 mL total volume). Eluted proteins were concentrated to 200 µL and quantitated using the BCA protein assay kit. Protein extracts (200 µg) were reduced with DTT (10 mM final concentration) for 45 min at 50 °C.

### 4.4. Label-Free Liquid Chromatography with Tandem Mass Spectrometry

Protein and phosphoprotein-enriched extracts were processed following the filter-aided sample preparation (FASP) protocol [28,29]. Each sample (200 µg) was deposited in a Microcon-30 kDa centrifugal filter unit (Merck Millipore, Burlington, MA, USA) and washed two times by centrifugation at 14,000× *g* for 15 min with 200 µL of UA buffer (8 M urea, 0.1 M Tris/HCl, pH 8.5). Samples were carbamydomethylated in 100 µL of 50 mM iodoacetamide in UA buffer for 20 min, then washed three times in 100 µL UA buffer followed by three washes in 100 µL of 50 mM ammonium bicarbonate in water. Filters were incubated with sequence grade trypsin (Promega, Madison, WI, USA) for 16 h at 37 °C using a protein:trypsin ratio of 50:1. After acidification with trifluoracetic acid and desalting on C18 tips (Zip-Tip C18 micro, Merck Millipore, Burlington, MA, USA), peptide samples were vacuum concentrated, reconstituted in HPLC buffer A (0.1% formic acid) and separated on a Dionex UltiMate 3000 HPLC System with an Easy Spray PepMap RSLC C18 column (250 mm, internal diameter of 75 µm) (Thermo Fisher Scientific, Rodano, Italy), adopting a five step acetonitrile (ACN)/formic acid gradient (5% ACN in 0.1% formic acid for 5 min, 5–35% ACN in 0.1% formic acid for 139 min, 35–60% ACN in 0.1% formic for 40 min, 60–100% ACN for 1 min, 100% ACN for 10 min, at a flow rate of 0.3 µL/min), and electrosprayed into an Orbitrap Fusion Tribrid (Thermo Fisher Scientific, Rodano, Italy) mass spectrometer. The LTQ-Orbitrap was operated in a positive mode in data-dependent acquisition mode to automatically alternate between a full scan (350–2000 m/z) in the orbitrap (at resolution 60,000, AGC target 1,000,000) and subsequent CID MS/MS in the linear ion trap of the 20 most intense peaks from full scan (normalized collision energy of 35%, 10 ms activation). Isolation window: 3 Da, unassigned charge states: rejected, charge state 1: rejected, charge states 2+, 3+, 4+: not rejected; dynamic exclusion enabled (60 s, exclusion list size: 200). Mass spectra were analyzed using MaxQuant software (Max-Planck-Institute of Biochemistry, Munich, Germany, version 1.6.3.3). The initial maximum allowed for mass deviation was set to 6 ppm for monoisotopic precursor ions and 0.5 Da for MS/MS peaks. Enzyme specificity was set to trypsin/P, and a maximum of two missed cleavages was allowed. Carbamidomethylation was set as a fixed modification, while N-terminal acetylation and methionine oxidation were set as variable modifications. The spectra were searched by the Andromeda search engine against the *Homo sapiens* Uniprot UP000005640 sequence database (79,684 proteins, released 23 February 2021). Protein identification required at least one unique or razor peptide per protein group. Quantification in MaxQuant was performed using the built-in extracted ion chromatogram (XIC)-based label-free quantification (LFQ) algorithm using fast LFQ. The required FDR was set to 1% at the peptide, 1% at the protein, and 1% at the site-modification level, and the minimum required peptide length was set to 7 amino acids. Statistical analyses were performed using the Perseus software (Max Planck Institute of Biochemistry, Munich, Germany, version 1.4.0.6). For each experimental group, every sample was run in technical triplicate and the proteins identified in at least 2 over 3 technical replicates were considered. For statistical analysis, ANOVA and Tukey post hoc test with a *p*-value threshold of 0.05 was applied. To exclude the presence of false positives from the analysis, Benjamini–Hochberg false discovery rate test was applied. 

The protein phosphorylation levels were calculated from the ratio between each of the three phosphoprotein values and the average total protein for every sample. To evaluate the presence of statistically significant differences in proteome, phosphoproteome, and phosphorylation levels, Student’s *t*-test was applied in each of the three analyzes, comparing the three values detected in the patient with the six values identified in the two young healthy controls, and the three values detected in the parent with the six values relative to the two adult controls. The results were considered statistically significant when *p* < 0.05.

### 4.5. Ingenuity Pathway Analysis

Functional and network analyses of statistically significant protein expression changes were performed through IPA software (Qiagen, Hilden, Germany). In brief, data sets with protein identifiers, statistical test *p*-values, and fold change values calculated from label-free LC-MS/MS experiments were analyzed by IPA. The “core analysis” function was used to interpret the data through the analysis of biological processes, canonical pathways, and upstream transcriptional regulators enriched with differentially regulated proteins. Then, the “comparison analysis” function was used to visualize and identify significant proteins or regulators across experimental conditions. *p*-values were calculated using a right-tailed Fisher’s exact test. The activation z-score was used to predict the activation/inhibition of a pathway/function/regulator [30]. A Fisher’s exact test *p*-value < 0.05 and a z-score ≤ −2 and ≥ 2, which takes into account the directionality of the effect observed, were considered statistically significant.

### 4.6. RAS Pathway Analysis

The proteins identified by proteome and phosphoproteome analysis were compared with those belonging to the RAS pathway list, provided by the National Cancer Institute (Table S5), to which two genes, *LRP1* and *LZTR1*, were added, as they regulate the RAS signaling pathway, even if not present in the list.

### 4.7. Immunoblotting

An aliquot (50 μg) of total protein extract for each sample prepared for proteomic analysis was loaded and resolved on 16% polyacrylamide gels. Analysis was performed in duplicate. Blots were incubated with PEBP1(RKIP) Polyclonal Antibody (Thermo Fisher Scientific, Rodano, Italy; #36-0700, 1:1000), RHOA Monoclonal Antibody (1B8-1C7) (Thermo Fisher Scientific, Rodano, Italy; #MA1-134, 1:1000), ERK1/ERK2 (MAPK1/3) Polyclonal Antibody (Thermo Fisher Scientific, Rodano, Italy; #44-654G, 1:1000), phospho-RKIP (Ser153) Polyclonal Antibody (Bioss, Woburn, MA, USA; bs-7075R, 1:1000), Phospho-RHOA (Ser188) Polyclonal Antibody (Thermo Fisher Scientific, Rodano, Italy; #PA5-104931, 1:1000), and Phospho-ERK1/ERK2 (MAPK1/3) (Thr202, Tyr204) Polyclonal Antibody (Thermo Fisher Scientific, Rodano, Italy; #36-8800, 1:1000). After washing, membranes were incubated with anti-rabbit (Seracare/KPL, Milford, MA, USA; 5220-0336, 1:10,000) or anti-mouse (Jackson Immunoresearch, Ely, UK; 715-035-151, 1:5000) secondary antibody conjugated with horseradish peroxidase. Signals were visualized by chemiluminescence using the ECL Prime detection kit and the Image Quant LAS 4000 (GE Healthcare, Little Chalfont, Buckinghamshire, UK) analysis system. Band quantification was performed using the Image Quant TL (GE Healthcare, Little Chalfont, Buckinghamshire, UK) software. The ratio of phosphorylated over the total protein band intensity after Western blot was assessed and differences between samples and their respective controls evaluated by statistical analysis (*t*-test, n = 2, *p*-value < 0.05).

## Figures and Tables

**Table 1:** Canonical pathways heat map displaying the most significant results (ordered by decreasing z-scores in patient 67) resulting from an IPA comparison analysis across different datasets.

Canonical Pathways	53 vs. yHC z-Score	67 vs. yHC z-Score	53F vs. aHC z-Score	53M vs. aHC z-Score	67F vs. aHC z-Score	67M vs. aHC z-Score	yHC vs. aHC z-Score
RHOA Signaling	1.89	3.051	N/A	1.342	N/A	1	2.236
CD28 Signaling in T Helper Cells	2.449	3	N/A	1	0	N/A	1
Signaling by Rho Family GTPases	2.121	2.84	N/A	0.816	0.447	1.633	1.89
Remodeling of Epithelial Adherens Junctions	2	2.646	N/A	N/A	N/A	N/A	N/A
fMLP Signaling in Neutrophils	2.449	2.333	N/A	N/A	N/A	N/A	N/A
CDC42 Signaling	2	2.333	N/A	N/A	N/A	N/A	N/A
Synaptogenesis Signaling Pathway	2.121	2.309	N/A	0.447	−1.633	N/A	0

The orange and blue colored rectangles indicate predicted pathway activation or predicted inhibition, respectively, via the z-score statistic (significant z-scores ≥ 2, ≤−2). Darker orange corresponds to more significant z-scores. The numbers 53 and 67, patients; F, father; M, mother; yHC, young healthy controls; aHC, adult healthy controls; N/A, not applicable.

## Data Availability

LC-MS/MS proteomics data are available in Dataverse (https://doi.org/10.13130/RD_UNIMI/PKZ7FJ, deposited on 30 October 2022).

## Supplementary Materials

The following supporting information can be downloaded at: https://www.mdpi.com/article/10.3390/ijms24044035/s1.
