# Supplementary material for: Changes of RAS Pathway Phosphorylation in Lymphoblastoid Cell Lines from Noonan Syndrome Patients Carrying Hypomorphic Variants in Two NS Genes
Source: Int J Mol Sci. 2023 Feb 17;24(4):4035. doi: 10.3390/ijms24044035 (PMC9959625; doi:10.3390/ijms24044035)
Supplement: Supplementary file 1 [file ijms-24-04035-s001.zip › ijms-2041318-supplementary/Legends to supplementary figures.pdf]

Legends to supplementary figures

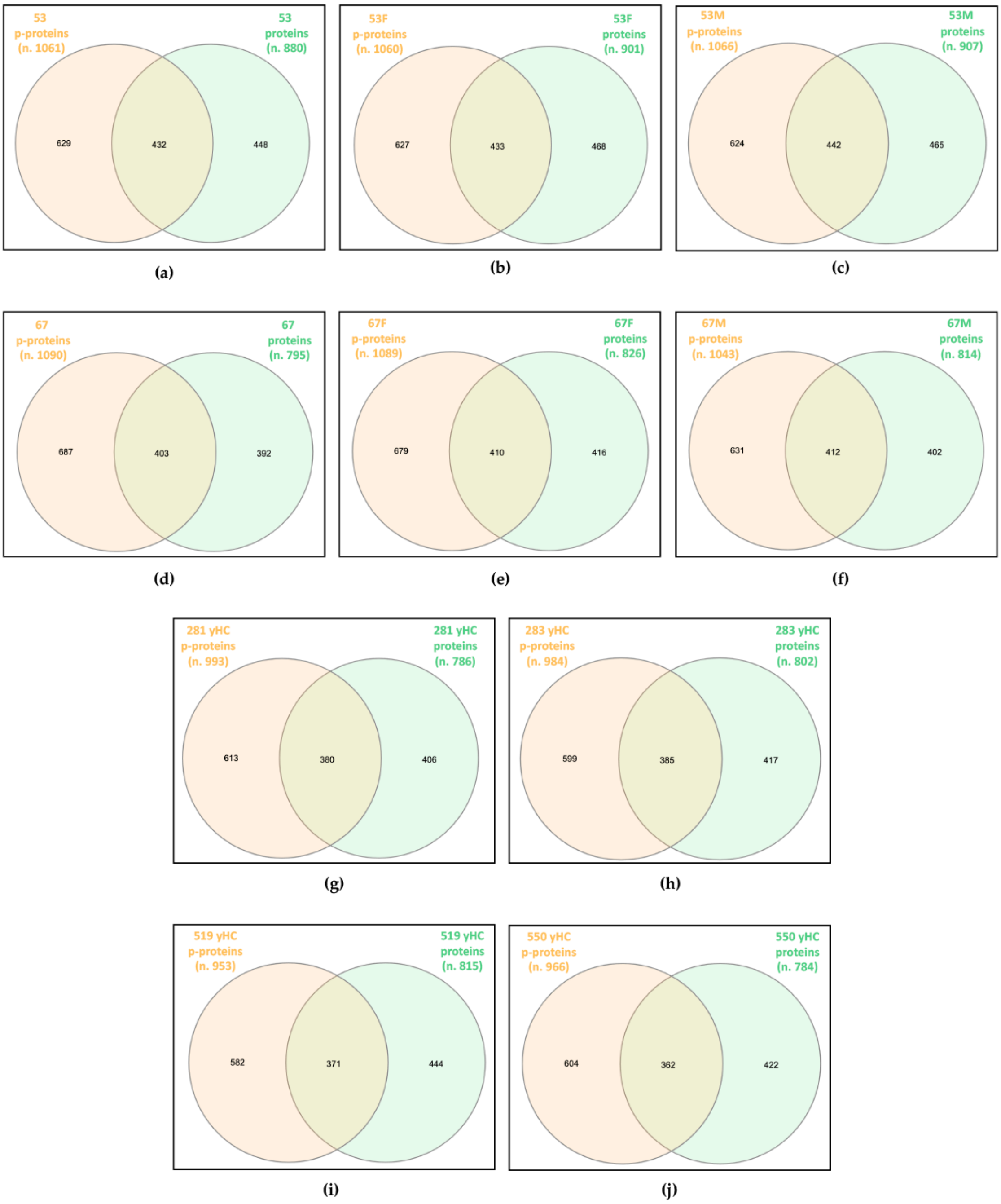

Figure S1. Proteins represented both in proteome and phosphoproteome analyses. The Venn diagrams, drawn using InteractiVenn tool, show the number of proteins identified by phosphoproteome analysis (p-proteins), proteome analysis (proteins), and in common between the two datasets, in: (a) Patient 53 (53); (b) Patient 53's father (53F); (c) Patient 53's mother (53M); (d) Patient 67 (67); (e) Patient 67's father (67F); (f) Patient 67's mother (67M); (g) 281 young healthy control (281 yHC); (h) 283 young healthy control (283 yHC); (i) 519 adult healthy control (519 aHC); (j) 550 adult healthy control (550 aHC).

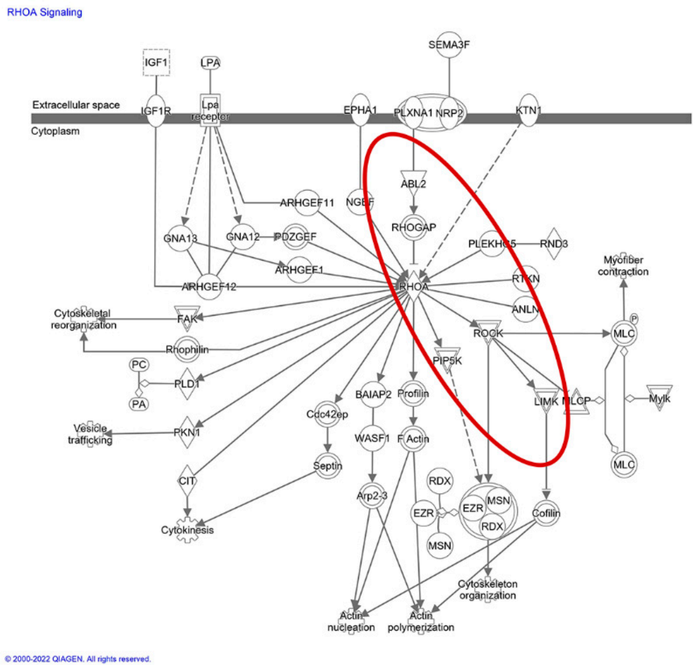
RHOA Signaling
SEMA3F
IGF1
LPA
Extracellular space
Cytoplasm
IGF1R
Lpa receptor
EPHA1
PLXNA1
NRP2
KTN1
ABL2
RHOGAP
ARHGEF11
NGEF
GNA13
GNA12
PDZGEF
ARHGEF1
ARHGEF12
PLEKHG5
RND3
Myofiber contraction
RHOA
RTKN
ANLN
Cytoskeletal reorganization
FAK
Rhophilin
ROCK
MLC
P
PIP5K
PC
PLD1
PA
BAIAP2
Profilin
LIMK
MLCP
Mylk
Vesicle trafficking
PKN1
Cdc42ep
WASF1
F Actin
Septin
MLC
CIT
RDX
Arp2-3
EZR
EZR
MSN
RDX
Cofilin
Cytokinesis
MSN
Cytoskeleton organization
Actin nucleation
Actin polymerization
© 2000-2022 QIAGEN. All rights reserved.

(a)

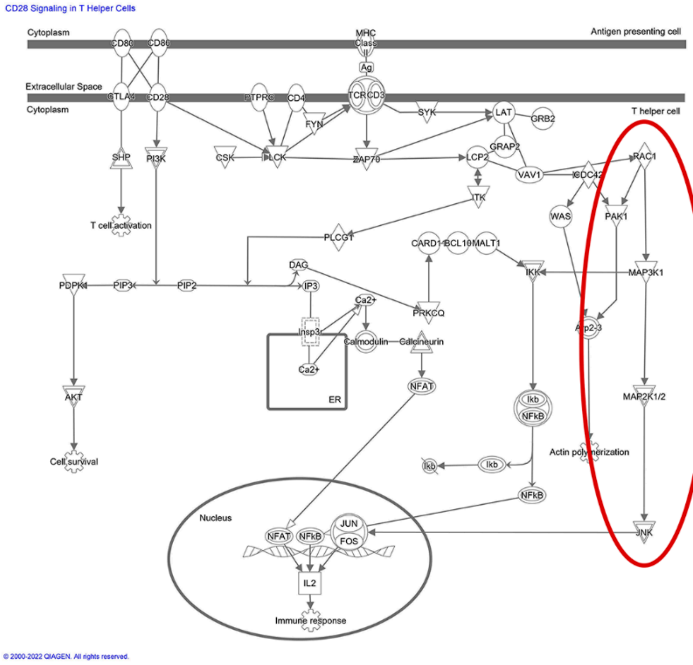
CD28 Signaling in T Helper Cells
Cytoplasm
CD80
CD86
MHC Class II
Antigen presenting cell
Ag
Extracellular Space
CTLA4
CD28
PTPRC
CD4
TCR
CD3
Cytoplasm
T helper cell
FYN
SYK
LAT
GRB2
SHP
PI3K
CSK
LCK
ZAP70
LCP2
GRAP2
VAV1
CDC42
RAC1
ITK
WAS
PAK1
T cell activation
PLCG1
CARD11
BCL10
MALT1
DAG
IKK
MAP3K1
PDPK1
PIP3
PIP2
IP3
Ca2+
PRKCQ
Arp2-3
Insp3r
Calmodulin
Calcineurin
Ca2+
ER
NFAT
AKT
Ikb
NFkB
MAP2K1/2
Ikb
Ikb
Actin polymerization
Cell survival
NFkB
Nucleus
JUN
FOS
NFAT
NFkB
JNK
IL2
Immune response
© 2000-2022 QIAGEN. All rights reserved.

(b)

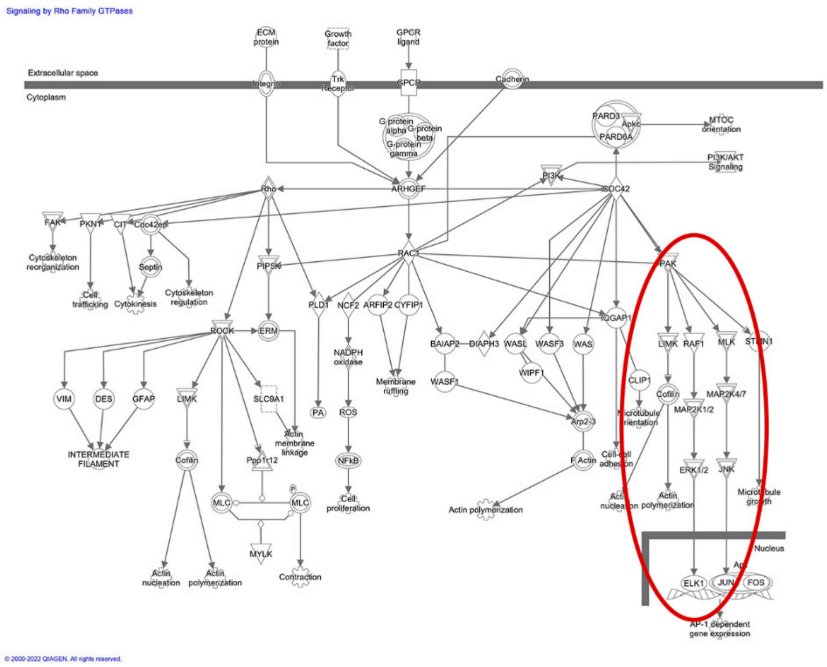
Signaling by Rho Family GTPases
ECM protein
Growth factor
GPCR ligand
Extracellular space
Cytoplasm
Integrin
Trk Receptor
GPCR
Cadherin
G-protein alpha
G-protein beta
G-protein gamma
PARD3
Apkc
PARD6A
MTOC orientation
PI3K/AKT Signaling
PI3K
CDC42
Rho
ARHGEF
FAK
PKN1
CIT
Cdc42ep
Cytoskeleton reorganization
Septin
Cell trafficking
Cytokinesis
Cytoskeleton regulation
RAC1
PIP5K
PAK
PLD1
NCF2
ARFIP2
CYFIP1
IQGAP1
ROCK
ERM
BAIAP2
DIAPH3
WASL
WASF3
WAS
LIMK
RAF1
MLK
STMN1
NADPH oxidase
Membrane ruffling
WASF1
WIPF1
CLIP1
Cofilin
MAP2K4/7
VIM
DES
GFAP
SLC9A1
PA
ROS
Arp2-3
MAP2K1/2
Microtubule orientation
INTERMEDIATE FILAMENT
Actin membrane linkage
Ppp1r12
NFkB
F Actin
Cell-cell adhesion
ERK1/2
JNK
MLC
Cell proliferation
Actin polymerization
Actin nucleation
Microtubule growth
MYLK
Contraction
Nucleus
ELK1
JUN
FOS
AP-1 dependent gene expression
© 2000-2022 QIAGEN. All rights reserved.

(c)

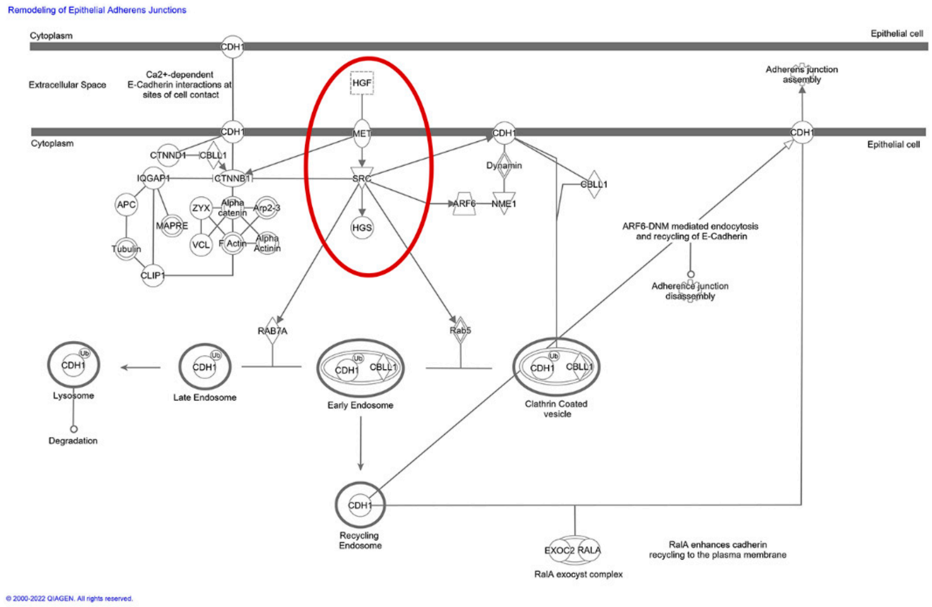
Remodeling of Epithelial Adherens Junctions
Cytoplasm
Epithelial cell
CDH1
Ca2+-dependent E-Cadherin interactions at sites of cell contact
Extracellular Space
HGF
Adherens junction assembly
MET
CTNND1
CBLL1
IQGAP1
CTNNB1
SRC
Dynamin
APC
ZYX
Alpha catenin
Arp2-3
MAPRE
ARF6
NME1
HGS
Tubulin
VCL
F Actin
Alpha Actinin
CLIP1
ARF6-DNM mediated endocytosis and recycling of E-Cadherin
Adherence junction disassembly
RAB7A
Rab5
Ub
Lysosome
Late Endosome
Early Endosome
Clathrin Coated vesicle
Degradation
Recycling Endosome
EXOC2
RALA
RalA exocyst complex
RalA enhances cadherin recycling to the plasma membrane
© 2000-2022 QIAGEN. All rights reserved.

(d)

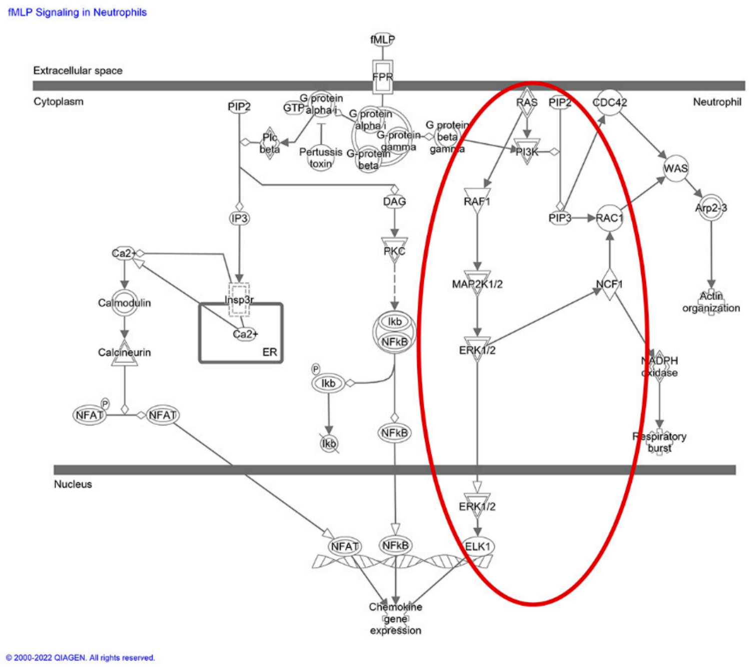
fMLP Signaling in Neutrophils
fMLP
FPR
Extracellular space
Cytoplasm
Neutrophil
PIP2
GTP
G protein alpha i
Plc beta
Pertussis toxin
G protein alpha i
G protein gamma
G protein beta
G protein beta gamma
RAS
PIP2
CDC42
PI3K
WAS
RAF1
PIP3
RAC1
Arp2-3
DAG
IP3
Ca2+
PKC
Calmodulin
Insp3r
Ca2+
ER
MAP2K1/2
NCF1
Actin organization
Ikb
NFkB
Calcineurin
ERK1/2
NADPH oxidase
P
Ikb
NFAT
NFAT
NFkB
Ikb
Respiratory burst
Nucleus
ERK1/2
NFAT
NFkB
ELK1
Chemokine gene expression
© 2000-2022 QIAGEN. All rights reserved.

(e)

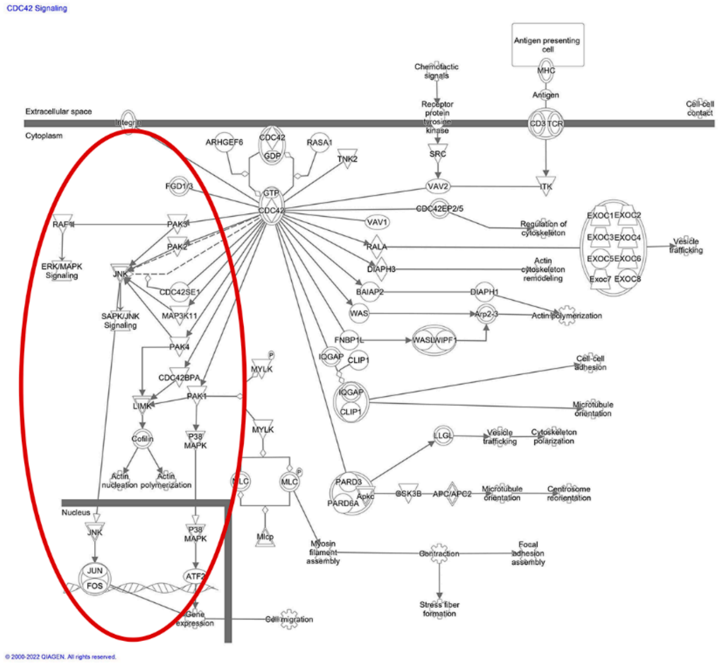
CDC42 Signaling
Antigen presenting cell
Chemotactic signals
MHC
Antigen
Receptor protein tyrosine kinase
Cell-cell contact
Extracellular space
Integrin
CD3 TCR
Cytoplasm
ARHGEF6
CDC42
GDP
RASA1
TNK2
SRC
FGD1/3
GTP
CDC42
VAV2
ITK
CDC42EP2/5
RAF1
PAK3
VAV1
Regulation of cytoskeleton
EXOC1
EXOC2
EXOC3
EXOC4
EXOC5
EXOC6
Exoc7
EXOC8
Vesicle trafficking
PAK2
RALA
ERK/MAPK Signaling
JNK
DIAPH3
Actin cytoskeleton remodeling
CDC42SE1
BAIAP2
DIAPH1
SAPK/JNK Signaling
MAP3K11
WAS
Arp2-3
Actin polymerization
FNBP1L
WASL WIPF1
PAK4
IQGAP
CLIP1
MYLK
Cell-cell adhesion
CDC42BPA
PAK1
IQGAP
CLIP1
LIMK
Microtubule orientation
MYLK
Cofilin
P38 MAPK
LLGL
Vesicle trafficking
Cytoskeleton polarization
Actin nucleation
Actin polymerization
MLC
MLC
PARD3
Apkc
PARD6A
GSK3B
APC/APC2
Microtubule orientation
Centrosome reorientation
Nucleus
JNK
P38 MAPK
Mlcp
Myosin filament assembly
Contraction
Focal adhesion assembly
JUN
FOS
ATF2
Stress fiber formation
Gene expression
Cell migration
© 2000-2022 QIAGEN. All rights reserved.

(f)

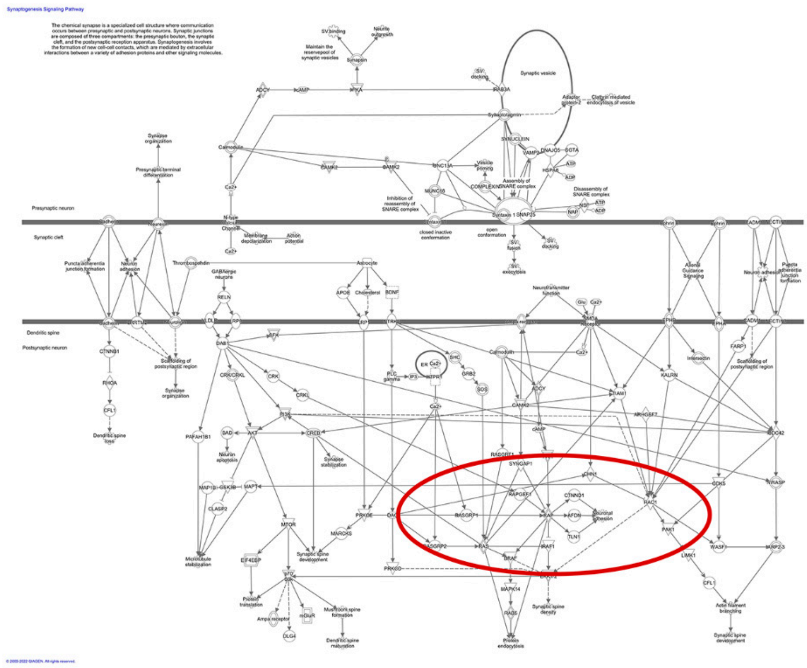

(g)

Figure S2. Main activated pathways highlighted by IPA enrichment analysis. Analysis of changed phosphoproteins/total protein datasets by IPA software identified the following seven canonical pathways significantly changed (Fisher's right-tailed exact test p-value < 0.05 and z-score ≥ 2 or ≤ −2) in patients 53 and 67 vs. young healthy controls: (a) RHOA Signaling; (b) CD28 Signaling in T Helper Cells; (c) Signaling by Rho Family GTPases; (d) Remodeling of Epithelial Adherens Junctions; (e) fMLP Signaling in Neutrophils; (f) CDC42 Signaling; (g) Synaptogenesis Signaling Pathway. In both patient's parents (53F-53M, 67F-67M) vs. age-matched controls, the same pathways were unchanged or only slightly activated. The pathways' branches connected to RAS signaling are circled in red.

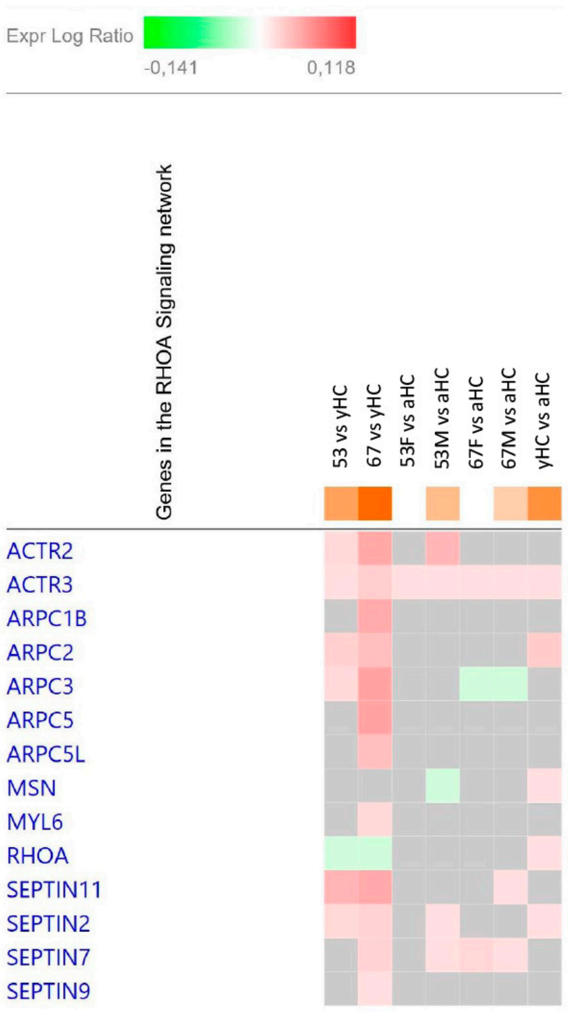

Figure S3. RHOA Signaling gene heatmap. The heatmap generated by IPA software shows the hypo-phosphorylation (in green) or hyper- phosphorylation (in red) of proteins belonging to RHOA signaling, based on log ratio data from LC-MS/MS analysis. Even if RHOA is hypo-phosphorylated in both patients (53 and 67) compared to young heathy controls (yHC), IPA software predicts the activation (orange rectangles) of RHOA Signaling in patients. In both patients' mothers (53M and 67M) vs. adult controls (aHC), the same pathway is only slightly activated, while RHOA Signaling activation is not predicted in the fathers (53F and 67F).
