# Supplementary material for: Changes of RAS Pathway Phosphorylation in Lymphoblastoid Cell Lines from Noonan Syndrome Patients Carrying Hypomorphic Variants in Two NS Genes
Source: Int J Mol Sci. 2023 Feb 17;24(4):4035. doi: 10.3390/ijms24044035 (PMC9959625; doi:10.3390/ijms24044035)
Supplement: Supplementary file 1 [file ijms-24-04035-s001.zip › ijms-2041318-supplementary/Table_S1.pdf]

Table S1. Changes in phosphoproteome expression pattern of families 53 and 67

Samples	Number of identified phosphoproteins	Number of phosphoproteins without altered expression level (%)	Number of phosphoproteins with increased expression level (%)	Number of phosphoproteins with decreased expression level (%)
53 (vs yHCs)	894	578 (64.7%)	153 (17.1%)	163 (18.2%)
53F (vs aHCs)	868	470 (54.1%)	135 (15.6%)	263 (30.3%)
53M (vs aHCs)	859	416 (48.4%)	129 (15.0%)	314 (36.6%)
67 (vs yHCs)	891	540 (60.6%)	175 (19.6%)	176 (19.8%)
67F (vs aHCs)	884	614 (69.5%)	130 (14.7%)	140 (15.8%)
67M (vs aHCs)	860	472 (54.9%)	156 (18.1%)	232 (27.0%)

53 and 67, patients; F, father; M, mother; yHCs, young healthy controls; aHCs, adult healthy controls.
