# Supplementary material for: Changes of RAS Pathway Phosphorylation in Lymphoblastoid Cell Lines from Noonan Syndrome Patients Carrying Hypomorphic Variants in Two NS Genes
Source: Int J Mol Sci. 2023 Feb 17;24(4):4035. doi: 10.3390/ijms24044035 (PMC9959625; doi:10.3390/ijms24044035)
Supplement: Supplementary file 1 [file ijms-24-04035-s001.zip › ijms-2041318-supplementary/Table_S2.pdf]

Table S2. Changes in phosphorylation degree of families 53 and 67 proteome

Samples	Number of identified phosphoproteins /proteins	Number of phosphoproteins without altered phosphorylation (%)	Number of phosphoproteins with increased phosphorylation (%)	Number of phosphoproteins with decreased phosphorylation (%)
53 (vs yHCs)	270	120 (44.4%)	106 (39.3%)	44 (16.3%)
53F (vs aHCs)	254	148 (58.3%)	49 (19.3%)	57 (22.4%)
53M (vs aHCs)	256	140 (54.7%)	43 (16.8%)	73 (28.5%)
67 (vs yHCs)	343	174 (50.7%)	114 (33.2%)	55 (16.1%)
67F (vs aHCs)	335	196 (58.5%)	71 (21.2%)	68 (20.3%)
67M (vs aHCs)	338	194 (57.4%)	71 (21.0%)	73 (21.6%)
yHCs (vs aHCs)	327	166 (50.8%)	95 (29.0%)	66 (20.2%)

53 and 67, patients; F, father; M, mother; yHCs, young healthy controls; aHCs, adult healthy controls.
