# Supplementary material for: Changes of RAS Pathway Phosphorylation in Lymphoblastoid Cell Lines from Noonan Syndrome Patients Carrying Hypomorphic Variants in Two NS Genes
Source: Int J Mol Sci. 2023 Feb 17;24(4):4035. doi: 10.3390/ijms24044035 (PMC9959625; doi:10.3390/ijms24044035)
Supplement: Supplementary file 1 [file ijms-24-04035-s001.zip › ijms-2041318-supplementary/Table_S3.pdf]

Table S3. Changes in proteome expression pattern of families 53 and 67

Samples	Number of identified proteins	Number of proteins without altered expression level (%)	Number of proteins with increased expression level (%)	Number of proteins with decreased expression level (%)
53 (vs yHCs)	566	301 (53.2%)	72 (12.7%)	193 (34.1%)
53F (vs aHCs)	578	295 (51.0%)	139 (24.1%)	144 (24.9%)
53M (vs aHCs)	585	284 (48.5%)	145 (24.8%)	156 (26.7%)
67 (vs yHCs)	732	328 (44.8%)	132 (18.0%)	272 (37.2%)
67F (vs aHCs)	752	453 (60.2%)	147 (19.6%)	152 (20.2%)
67M (vs aHCs)	748	489 (65.4%)	134 (17.9%)	125 (16.7%)

53 and 67, patients; F, father; M, mother; yHCs, young healthy controls; aHCs, adult healthy controls.
