# Supplementary material for: Changes of RAS Pathway Phosphorylation in Lymphoblastoid Cell Lines from Noonan Syndrome Patients Carrying Hypomorphic Variants in Two NS Genes
Source: Int J Mol Sci. 2023 Feb 17;24(4):4035. doi: 10.3390/ijms24044035 (PMC9959625; doi:10.3390/ijms24044035)
Supplement: Supplementary file 1 [file ijms-24-04035-s001.zip › ijms-2041318-supplementary/Table_S4.pdf]

Table S4. Changes in phosphorylation degree of dysregulated proteins in families 53 and 67

Samples	Number of proteins with altered expression and phosphorylation	Number of hyper-expressed and hyper-phosphorylated proteins (%)	Number of hypo-expressed and hypo-phosphorylated proteins (%)	Number of hyper-expressed and hypo-phosphorylated proteins (%)	Number of hypo-expressed and hyper-phosphorylated proteins (%)
53 (vs yHCs)	83	3 (3.6%)	6 (7.2%)	12 (14.5%)	62 (74.7%)
53F (vs aHCs)	67	7 (10.5%)	10 (14.9%)	26 (38.8%)	24 (35.8%)
53M (vs aHCs)	68	8 (11.8%)	8 (11.8%)	29 (42.6%)	23 (33.8%)
67 (vs yHCs)	109	8 (7.3%)	6 (5.5%)	23 (21.1%)	72 (66.1%)
67F (vs aHCs)	78	5 (6.4%)	6 (7.7%)	35 (44.9%)	32 (41.0%)
67M (vs aHCs)	65	5 (7.7%)	8 (12.3%)	26 (40.0%)	26 (40.0%)

53 and 67, patients; F, father; M, mother; yHCs, young healthy controls; aHCs, adult healthy controls.
