# Supplementary material for: Changes of RAS Pathway Phosphorylation in Lymphoblastoid Cell Lines from Noonan Syndrome Patients Carrying Hypomorphic Variants in Two NS Genes
Source: Int J Mol Sci. 2023 Feb 17;24(4):4035. doi: 10.3390/ijms24044035 (PMC9959625; doi:10.3390/ijms24044035)
Supplement: Supplementary file 1 [file ijms-24-04035-s001.zip › ijms-2041318-supplementary/Table_S5.pdf]

Table S5. RAS pathway genes

Gene symbol	Protein name from BioGPS	Alternative gene names from BioGPS
AKT1	RAC-alpha serine/threonine-protein kinase	AKT, CWS6, PKB, PKB-ALPHA, PRKBA, RAC, RAC-ALPHA
AKT2	RAC-beta serine/threonine-protein kinase	HIHGHH, PKBB, PKBBETA, PRKBB, RAC-BETA
AKT3	RAC-gamma serine/threonine-protein kinase	MPPH, MPPH2, PKB-GAMMA, PKBG, PRKBG, RAC-PK-gamma, RAC-gamma, STK-2
ALK	anaplastic lymphoma receptor tyrosine kinase; ALK tyrosine kinase receptor	CD246, NBLST3
APAF1	apoptotic peptidase activating factor 1	APAF-1, CED4
ARAF	serine/threonine-protein kinase A-Raf	A-RAF, ARAF1, PKS2, RAFA1
ARHGAP35	Rho GTPase-activating protein 35	GRF-1, GRLF1, P190-A, P190A, p190ARhoGAP, p190RhoGAP
ARHGEF2	Rho/Rac guanine nucleotide exchange factor (GEF) 2	GEF, GEF-H1, GEFH1, LFP40, P40
BARD1	BRCA1 associated RING domain 1	[none]
BRAF	serine/threonine-protein kinase B-raf	B-RAF1, BRAF1, NS7, RAFB1
BRCA1	breast cancer 1, early onset	BRCAI, BRCC1, BROVCA1, IRIS, PNCA4, PPP1R53, PSCP, RNF53
BRCA2	breast cancer 2, early onset	BRCC2, BROVCA2, FACD, FAD, FAD1, FANCD, FANCD1, GLM3, PNCA2
BRIP1	BRCA1 interacting protein C-terminal helicase 1	BACH1, FANCJ, OF
BUB1	BUB1 mitotic checkpoint serine/threonine kinase	BUB1A, BUB1L, hBUB1
CASP3	caspase 3, apoptosis-related cysteine peptidase	CPP32, CPP32B, SCA-1
CASP7	caspase 7, apoptosis-related cysteine peptidase	CASP-7, CMH-1, ICE-LAP3, LICE2, MCH3
CASP8	caspase 8, apoptosis-related cysteine peptidase	ALPS2B, CAP4, Casp-8, FLICE, MACH, MCH5
CBL	Cbl proto-oncogene, E3 ubiquitin protein ligase	C-CBL, CBL2, FRA11B, NSLL, RNF55
CBLB	Cbl proto-oncogene B, E3 ubiquitin protein ligase	Cbl-b, RNF56
CBLC	Cbl proto-oncogene C, E3 ubiquitin protein ligase	CBL-3, CBL-SL, RNF57
CCNA1	cyclin A1	CT146
CCNA2	cyclin A2	CCN1, CCNA
CCND1	cyclin D1	BCL1, D11S287E, PRAD1, U21B31
CCND2	cyclin D2	KIAK0002, MPPH3
CCND3	cyclin D3	[none]
CDC25A	cell division cycle 25A	CDC25A2
CDC6	cell division cycle 6	cell division cycle 6
CDK2	cyclin-dependent kinase 2	CDKN2, p33(CDK2)
CDK4	cyclin-dependent kinase 4	CMM3, PSK-J3
CDK6	cyclin-dependent kinase 6	PLSTIRE
CDKN1A	cyclin-dependent kinase inhibitor 1A (p21, Cip1)	CAP20, CDKN1, CIP1, MDA-6, P21, SDI1, WAF1, p21CIP1
CNKSR1	connector enhancer of kinase suppressor of Ras 1	CNK, CNK1
CNKSR2	connector enhancer of kinase suppressor of Ras 2	CNK2, KSR2, MAGUIN
CYTH2	cytohesin 2	ARNO, CTS18, CTS18.1, PSCD2, PSCD2L, SEC7L, Sec7p-L, Sec7p-like
DAB2IP	DAB2 interacting protein	AF9Q34, AIP-1, AIP1, DIP1/2
DHFR	dihydrofolate reductase	DHFRP1, DYR

DUSP1	dual specificity phosphatase 1	CL100, HVH1, MKP-1, MKP1, PTPN10
DUSP2	dual specificity phosphatase 2	PAC-1, PAC1
DUSP3	dual specificity phosphatase 3	VHR
DUSP4	dual specificity phosphatase 4	HVH2, MKP-2, MKP2, TYP
DUSP5	dual specificity phosphatase 5	DUSP, HVH3
DUSP6	dual specificity phosphatase 6	HH19, MKP3, PYST1
E2F1	E2F transcription factor 1	E2F-1, RBAP1, RBBP3, RBP3
E2F2	E2F transcription factor 2	E2F-2
E2F3	E2F transcription factor 3	E2F-3
E2F7	E2F transcription factor 7	[none]
ECT2	epithelial cell transforming 2	ARHGEF31
EGFR	epidermal growth factor receptor	ERBB, ERBB1, HER1, PIG61, mENA
EIF4EBP1	eukaryotic translation initiation factor 4E-binding protein 1	4E-BP1, 4EBP1, BP-1, PHAS-I
EIF4EBP2	eukaryotic translation initiation factor 4E binding protein 2	4EBP2, PHASII
EIF4EBP3	eukaryotic translation initiation factor 4E binding protein 3	4E-BP3, 4EBP3
ELK1	ELK1, member of ETS oncogene family	[none]
ERBB2	receptor tyrosine-protein kinase erbB-2	CD340, HER-2, HER-2/neu, HER2, MLN 19, NEU, NGL, TKR1
ERF	Ets2 repressor factor	CRS4, PE-2, PE2
ETS1	v-ets avian erythroblastosis virus E26 oncogene homolog 1	ETS-1, EWSR2, p54
ETS2	v-ets avian erythroblastosis virus E26 oncogene homolog 2	ETS2IT1
EXOC1	Exocyst complex component 1	BM-102, SEC3, SEC3L1, SEC3P
EXOC2	exocyst complex component 2	SEC5, SEC5L1, Sec5p
EXOC3	exocyst complex component 3	SEC6, SEC6L1, Sec6p
EXOC4	exocyst complex component 4	SEC8, SEC8L1, Sec8p
EXOC5	exocyst complex component 5	HSEC10, PRO1912, SEC10, SEC10L1, SEC10P
EXOC6	exocyst complex component 6	EXOC6A, SEC15, SEC15L, SEC15L1, SEC15L3, Sec15p
EXOC7	exocyst complex component 7	2-5-3p, EX070, EXO70, EXOC1, Exo70p, YJL085W
EXOC8	exocyst complex component 8	EXO84, Exo84p, SEC84
FANCA	Fanconi anemia, complementation group A	FA, FA-H, FA1, FAA, FACA, FAH, FANCH
FANCC	Fanconi anemia, complementation group C	FA3, FAC, FACC
FGFR1	fibroblast growth factor receptor 1	BFGFR, CD331, CEK, FGFBR, FGFR-1, FLG, FLT-2, FLT2, HBGFR, HH2, HRTFDS, KAL2, N-SAM, OGD, bFGF-R-1
FGFR2	fibroblast growth factor receptor 2	BBDS, BEK, BFR-1, CD332, CEK3, CFD1, ECT1, JWS, K-SAM, KGFR, TK14, TK25
FGFR3	fibroblast growth factor receptor 3	CH, CD333, CEK2, HSFGFR3EX, JTK4
FGFR4	fibroblast growth factor receptor 4	CD334, JTK2, TKF
FLT3	fms-related tyrosine kinase 3	CD135, FLK-2, FLK2, STK1
FNTA	farnesyltransferase, CAAX box, alpha	FPTA, PGGT1A, PTAR2
FNTB	farnesyltransferase, CAAX box, beta	FPTB

FOS	FBJ murine osteosarcoma viral oncogene homolog	AP-1, C-FOS, p55
FOSL1	FOS-like antigen 1	FRA, FRA1, fra-1
GRB10	growth factor receptor-bound protein 10	GRB-IR, Grb-10, IRBP, MEG1, RSS
GRB2	growth factor receptor-bound protein 2	ASH, EGFRBP-GRB2, Grb3-3, MST084, MSTP084, NCKAP2
HRAS	GTPase HRas	C-BAS/HAS, C-H-RAS, C-HA-RAS1, CTLO, H-RASIDX, HAMSV, HRAS1, RASH1, p21ras
ICMT	isoprenylcysteine carboxyl methyltransferase	HSTE14, MST098, MSTP098, PCCMT, PCMT, PPMT
INPP4B	inositol polyphosphate-4-phosphatase, type II, 105kDa	[none]
INSR	insulin receptor	CD220, HHF5
INSRR	insulin receptor-related receptor	IRR
IRS1	insulin receptor substrate 1	HIRS-1
IRS2	insulin receptor substrate 2	IRS-2
JUN	Jun proto-oncogene	AP-1, AP1, c-Jun
KRAS	GTPase KRas	C-K-RAS, CFC2, K-RAS2A, K-RAS2B, K-RAS4A, K-RAS4B, KI-RAS, KRAS1, KRAS2, NS, NS3, RASK2
KSR1	kinase suppressor of Ras 1	KSR, RSU2
KSR2	kinase suppressor of Ras 2	[none]
LRP1	LDL receptor related protein 1	A2MR, APOER, APR, CD91, IGFBP-3R, IGFBP3R, IGFBP3R1, KPA, LRP, LRP1A, TGFBR5
LZTR1	leucine zipper like transcription regulator 1	BTBD29, LZTR-1, NS10, NS2, SWNTS2
MAP2K1	dual specificity mitogen-activated protein kinase kinase 1	CFC3, MAPKK1, MEK1, MKK1, PRKMK1
MAP2K2	dual specificity mitogen-activated protein kinase kinase 2	CFC4, MAPKK2, MEK2, MKK2, PRKMK2
MAPK1	mitogen-activated protein kinase 1	ERK, ERK-2, ERK2, ERT1, MAPK2, P42MAPK, PRKM1, PRKM2, p38, p40, p41, p41mapk, p42-MAPK
MAPK3	mitogen-activated protein kinase 3	ERK-1, ERK1, ERT2, HS44KDAP, HUMKER1A, P44ERK1, P44MAPK, PRKM3, p44-ERK1, p44-MAPK
MCM3	minichromosome maintenance complex component 3	HCC5, P1-MCM3, P1.h, RLFB
MCM4	minichromosome maintenance complex component 4	CDC21, CDC54, NKCD, NKGCD, P1-CDC21, hCdc21
MCM5	minichromosome maintenance complex component 5	CDC46, P1-CDC46
MCM6	minichromosome maintenance complex component 6	MCG40308, Mis5, P105MCM
MCM7	minichromosome maintenance complex component 7	CDC47, MCM2, P1.1-MCM3, P1CDC47, P85MCM, PNAS146, PPP1R104
MDM2	MDM2 proto-oncogene, E3 ubiquitin protein ligase	ACTFS, HDMX, hdm2
MET	MET proto-oncogene, receptor tyrosine kinase; hepatocyte growth factor receptor	AUTS9, HGFR, RCCP2, c-Met
MLST8	MTOR associated protein, LST8 homolog (S. cerevisiae)	GBL, GbetaL, LST8, POP3, WAT1
MRAS	muscle RAS oncogene homolog	M-RAs, R-RAS3, RRAS3
MTOR	mechanistic target of rapamycin (serine/threonine kinase)	FRAP, FRAP1, FRAP2, RAFT1, RAPT1
MYB	v-myb avian myeloblastosis viral oncogene homolog	Cmyb, c-myb, c-myb_CDS, efg
MYC	v-myc avian myelocytomatosis viral oncogene homolog	MRTL, MYCC, bHLHe39, c-Myc
NF1	neurofibromin	NFNS, VRNF, WSS

NFE2L2	nuclear factor, erythroid 2-like 2	NRF2
NFKB1	nuclear factor of kappa light polypeptide gene enhancer in B-cells 1	EBP-1, KBF1, NF-kB1, NF-kappa-B, NF-kappaB, NFKB-p105, NFKB-p50, NFkappaB, p105, p50
NOX1	NADPH oxidase 1	GP91-2, MOX1, NOH-1, NOH1
NRAS	GTPase NRas	ALPS4, CMNS, N-ras, NCMS, NRAS1, NS6
PAK1	p21 protein (Cdc42/Rac)-activated kinase 1	PAKalpha
PAK2	p21 protein (Cdc42/Rac)-activated kinase 2	PAK65, PAKgamma
PAK3	p21 protein (Cdc42/Rac)-activated kinase 3	CDKN1A, MRX30, MRX47, OPHN3, PAK3beta, bPAK, hPAK3
PAK4	p21 protein (Cdc42/Rac)-activated kinase 4	[none]
PDGFRA	platelet-derived growth factor receptor alpha	CD140A, PDGFR-2, PDGFR2, RHEPDGFRA
PDGFRB	platelet-derived growth factor receptor beta	CD140B, IBGC4, IMF1, JTK12, PDGFR, PDGFR-1, PDGFR1
PDPK1	3-phosphoinositide-dependent protein kinase 1	PDK1, PDPK2, PRO0461
PEA15	phosphoprotein enriched in astrocytes 15	HMAT1, HUMMAT1H, MAT1, MAT1H, PEA-15, PED
PEBP1	phosphatidylethanolamine binding protein 1	HCNP, HCNPpp, HEL-210, HEL-S-34, PBP, PEBP, PEBP-1, RKIP
PIK3CA	Phosphatidylinositol 4,5-bisphosphate 3-kinase catalytic subunit alpha isoform	CLOVE, CWS5, MCAP, MCM, MCMTC, PI3K, p110-alpha
PIK3CD	phosphatidylinositol-4,5-bisphosphate 3-kinase, catalytic subunit delta	APDS, IMD14, P110DELTA, PI3K, p110D
PIK3CG	phosphatidylinositol-4,5-bisphosphate 3-kinase, catalytic subunit gamma	PI3CG, PI3K, PI3Kgamma, PIK3, p110gamma, p120-PI3K
PIK3R1	phosphatidylinositol 3-kinase regulatory subunit alpha	AGM7, GRB1, p85, p85-ALPHA
PIK3R2	phosphatidylinositol 3-kinase regulatory subunit beta	MPPH, MPPH1, P85B, p85, p85-BETA
PIK3R3	phosphatidylinositol 3-kinase regulatory subunit gamma	p55, p55-GAMMA
PIK3R5	phosphoinositide 3-kinase regulatory subunit 5	F730038I15Rik, FOAP-2, P101-PI3K, p101
PIK3R6	phosphoinositide 3-kinase regulatory subunit 6	C17orf38, HsT41028, p84 PIKAP, p87(PIKAP), p87PIKAP
PIN1	peptidylprolyl cis/trans isomerase, NIMA-interacting 1	DOD, UBL5
PLCE1	phospholipase C, epsilon 1	NPHS3, PLCE, PPLC
PLXNB1	plexin B1	PLEXIN-B1, PLXN5, SEP
PPP1CA	protein phosphatase 1, catalytic subunit, alpha isozyme	PP-1A, PP1A, PP1alpha, PPP1A
PREX2	phosphatidylinositol-3,4,5-trisphosphate-dependent Rac exchange factor 2	6230420N16Rik, DEP.2, DEPDC2, P-REX2, PPP1R129
PRKAA1	5'-AMP-activated protein kinase catalytic subunit alpha-1	AMPK, AMPKa1
PRKAA2	5'-AMP-activated protein kinase catalytic subunit alpha-2	AMPK, AMPK2, AMPKa2, PRKAA
PRKAB1	5'-AMP-activated protein kinase subunit beta-1	AMPK, HAMPKb
PRKAB2	5'-AMP-activated protein kinase subunit beta-2	[none]
PRKAG1	protein kinase, AMP-activated, gamma 1 non-catalytic subunit	AMPKG
PRKAG2	protein kinase, AMP-activated, gamma 2 non-catalytic subunit	AAKG, AAKG2, CMH6, H91620p, WPWS
PRKAG3	protein kinase, AMP-activated, gamma 3 non-catalytic subunit	AMPKG3
PTEN	phosphatidylinositol 3,4,5-trisphosphate 3-phosphatase and dual-specificity protein phosphatase PTEN	10q23del, BZS, CWS1, DEC, GLM2, MHAM, MMAC1, PTEN1, TEP1

PTPN11	protein tyrosine phosphatase, non-receptor type 11	BPTP3, CFC, NS1, PTP-1D, PTP2C, SH-PTP2, SH-PTP3, SHP2
RAC1	Ras-related C3 botulinum toxin substrate 1 (rho family, small GTP binding protein Rac1)	Rac-1, TC-25, p21-Rac1
RAC2	Ras-related C3 botulinum toxin substrate 2 (rho family, small GTP binding protein Rac2)	EN-7, Gx, HSPC022, p21-Rac2
RAC3	Ras-related C3 botulinum toxin substrate 3 (rho family, small GTP binding protein Rac3)	[none]
RAD52	RAD52 homolog (S. cerevisiae)	[none]
RAF1	RAF proto-oncogene serine/threonine-protein kinase	CMD1NN, CRAF, NS5, Raf-1, c-Raf
RALA	Ras-related protein Ral-A	RAL
RALB	Ras-related protein Ral-B	[none]
RALBP1	RalA binding protein 1	RIP1, RLIP1, RLIP76
RALGAPA1	Ral GTPase activating protein, alpha subunit 1 (catalytic)	GARNL1, GRIPE, RalGAPalpha1, TULIP1, p240
RALGAPA2	Ral GTPase activating protein, alpha subunit 2 (catalytic)	AS250, C20orf74, bA287B20.1, dJ1049G11, dJ1049G11.4, p220
RALGDS	Ral guanine nucleotide dissociation stimulator	RGDS, RGF, RalGEF
RAPGEF1	Rap guanine nucleotide exchange factor (GEF) 1	C3G, GRF2
RAPGEF2	Rap guanine nucleotide exchange factor (GEF) 2	CNrasGEF, NRAPGEP, PDZ-GEF1, PDZGEF1, RA-GEF, RA-GEF-1, Rap-GEP, nRap GEP
RASA1	Ras GTPase-activating protein 1	CM-AVM, CMAVM, GAP, PKWS, RASA, RASGAP, p120GAP, p120RASGAP
RASA2	Ras GTPase-activating protein 2	GAP1M
RASA3	Ras GTPase-activating protein 3	GAP1IP4BP, GAPIII
RASAL1	RasGAP-activating-like protein 1	RASAL
RASAL2	Ras GTPase-activating protein nGAP	NGAP
RASAL3	RAS protein activator like-3	[none]
RASGRF1	Ras protein-specific guanine nucleotide-releasing factor 1	CDC25, CDC25L, GNRP, GRF1, GRF55, H-GRF55, PP13187, ras-GRF1
RASGRF2	Ras protein-specific guanine nucleotide-releasing factor 2	GRF2, RAS-GRF2
RASGRP1	RAS guanyl-releasing protein 1	CALDAG-GEFI, CALDAG-GEFII, RASGRP, V, hRasGRP1
RASGRP2	RAS guanyl-releasing protein 2	CALDAG-GEFI, CDC25L
RASGRP3	Ras guanyl-releasing protein 3	GRP3, KIAA0846
RASGRP4	RAS guanyl-releasing protein 4	[none]
RASSF1	Ras association (RalGDS/AF-6) domain family member 1	123F2, NORE2A, RASSF1A, RDA32, REH3P21
RASSF10	Ras association (RalGDS/AF-6) domain family (N-terminal) member 10	[none]
RASSF2	Ras association (RalGDS/AF-6) domain family member 2	CENP-34, RASFADIN
RASSF3	Ras association (RalGDS/AF-6) domain family member 3	RASSF5
RASSF4	Ras association (RalGDS/AF-6) domain family member 4	AD037
RASSF5	Ras association (RalGDS/AF-6) domain family member 5	Maxp1, NORE1, NORE1A, NORE1B, RAPL, RASSF3
RASSF6	Ras association (RalGDS/AF-6) domain family member 6	[none]
RASSF7	Ras association (RalGDS/AF-6) domain family (N-terminal) member 7	C11orf13, HRAS1, HRC1
RASSF8	Ras association (RalGDS/AF-6) domain family (N-terminal) member 8	C12orf2, HOJ1

RASSF9	Ras association (RalGDS/AF-6) domain family (N-terminal) member 9	P-CIP1, PAMCI, PCIP1
RB1	Retinoblastoma 1	OSRC, PPP1R130, RB, p105-Rb, pRb, pp110
RCE1	Ras converting CAAX endopeptidase 1	FACE2, RCE1A, RCE1B
RGL1	ral guanine nucleotide dissociation stimulator-like 1	RGL
RGL2	ral guanine nucleotide dissociation stimulator-like 2	HKE1.5, KE1.5, RAB2L
RGL3	ral guanine nucleotide dissociation stimulator-like 3	[none]
RHEB	Ras Homolog Enriched In Brain, GTP-binding protein Rheb	RHEB2
RHOA	Ras homolog family member A	ARH12, ARHA, RHO12, RHOH12
RHOB	Ras homolog family member B	ARH6, ARHB, MST081, MSTP081, RHOH6
RHOC	Ras homolog family member C	ARH9, ARHC, H9, RHOH9
ROCK1	Rho-associated, coiled-coil containing protein kinase 1	P160ROCK, ROCK-I
ROCK2	Rho-associated, coiled-coil containing protein kinase 2	ROCK-II
ROS1	ROS proto-oncogene 1, receptor tyrosine kinase	MCF3, ROS, c-ros-1
RPS6KA1	ribosomal protein S6 kinase alpha-1	MAPKAPK1A, RSK1
RPS6KA2	ribosomal protein S6 kinase alpha-2	HU-2, MAPKAPK1C, RSK, RSK3, S6K-alpha, S6K-alpha2, p90-RSK3, pp90RSK3
RPS6KA3	ribosomal protein S6 kinase alpha-3	CLS, HU-3, ISPK-1, MAPKAPK1B, MRX19, RSK, RSK2, S6K-alpha3, p90-RSK2, pp90RSK2
RPS6KA6	ribosomal protein S6 kinase alpha-6	PP90RSK4, RSK4
RPS6KB1	ribosomal protein S6 kinase, 70kDa, polypeptide 1	PS6K, S6K, S6K-beta-1, S6K1, STK14A, p70 S6KA, p70(S6K)-alpha, p70-S6K, p70-alpha
RPS6KB2	ribosomal protein S6 kinase, 70kDa, polypeptide 2	KLS, P70-beta, P70-beta-1, P70-beta-2, S6K-beta2, S6K2, SRK, STK14B, p70(S6K)-beta, p70S6Kb
RPTOR	regulatory associated protein of MTOR, complex 1	KOG1, Mip1
SAV1	salvador family WW domain containing protein 1	SAV, WW45, WWP4
SCRIB	scribbled planar cell polarity protein	CRIB1, SCRB1, SCRIB1, Vartul
SHC1	SHC (Src homology 2 domain containing) transforming protein 1	SHC, SHCA
SHC2	SHC (Src homology 2 domain containing) transforming protein 2	SCK, SHCB, SLI
SHC3	SHC (Src homology 2 domain containing) transforming protein 3	N-Shc, NSHC, RAI, SHCC
SHC4	SHC (Src homology 2 domain containing) transforming protein 4	RaLP, SHCD
SHOC2	Soc-2 suppressor of clear homolog (C. elegans)	SIAA0862, SOC2, SUR8
SOS1	Son of sevenless homolog 1	GF1, GGF1, GINGF, HGF, NS4
SOS2	Son of sevenless homolog 2	[none]
SPRED1	Sprouty-related, EVH1 domain-containing protein 1	NFLS, PPP1R147, hSpred1, spred-1
SPRED2	Sprouty-related, EVH1 domain-containing protein 2	Spred-2
SPRED3	Sprouty-related, EVH1 domain-containing protein 3	Eve-3, spred-3
SPRY1	protein sprouty homolog 1	hSPRY1
SPRY2	protein sprouty homolog 2	hSPRY2
SPRY3	protein sprouty homolog 3	HSPRY3, spry-3
SPRY4	protein sprouty homolog 4	HH17
STK11	serine/threonine-protein kinase STK11	LKB1, PJS, hLKB1

STK3	serine/threonine kinase 3	KRS1, MST2
STK4	serine/threonine kinase 4	KRS2, MST1, TIIAC, YSK3
TBK1	TANK-binding kinase 1	NAK, T2K
TFDP1	transcription factor Dp-1	DP1, DRTF1, Dp-1
TFDP2	transcription factor Dp-2 (E2F dimerization partner 2)	DP2
TIAM1	T-lymphoma invasion and metastasis-inducing protein 1	[none]
TIAM2	T-lymphoma invasion and metastasis-inducing protein 2	STEF, TIAM-2
TK1	thymidine kinase 1, soluble	TK2
TP53	tumor protein p53	BCC7, LFS1, P53, TRP53
TP73	tumor protein p73	P73
TSC1	Tuberous Sclerosis 1, Hamartin	LAM, TSC
TSC2	Tuberous Sclerosis 2, Tuberin	LAM, PPP1R160, TSC4
TYMS	thymidylate synthetase	HST422, TMS, TS
UNG	uracil-DNA glycosylase	DGU, HIGM4, HIGM5, UDG, UNG1, UNG15, UNG2
VAV1	vav 1 guanine nucleotide exchange factor	VAV
YAP1	Yes-associated protein 1	COB1, YAP, YAP2, YAP65, YKI
