# Supplementary material for: Changes of RAS Pathway Phosphorylation in Lymphoblastoid Cell Lines from Noonan Syndrome Patients Carrying Hypomorphic Variants in Two NS Genes
Source: Int J Mol Sci. 2023 Feb 17;24(4):4035. doi: 10.3390/ijms24044035 (PMC9959625; doi:10.3390/ijms24044035)
Supplement: Supplementary file 1 [file ijms-24-04035-s001.zip › ijms-2041318-supplementary/Table_S6.pdf]

Table S6. Differentially phosphorylated proteins belonging to RAS pathway

Samples	RAS pathway phosphoproteins with increased phosphorylation	RAS pathway phosphoproteins with decreased phosphorylation
53 (vs yHCs)	PEBP1, GRB2	RHOA
53F (vs aHCs)		
53M (vs aHCs)		PEBP1, GRB2
67 (vs yHCs)	GRB2	MAPK1, RHOA
67F (vs aHCs)		MAPK1, GRB2
67M (vs aHCs)		PEBP1

53 and 67, patients; F, father; M, mother; yHCs, young healthy controls; aHCs, adult healthy controls.
