# Supplementary material for: Changes of RAS Pathway Phosphorylation in Lymphoblastoid Cell Lines from Noonan Syndrome Patients Carrying Hypomorphic Variants in Two NS Genes
Source: Int J Mol Sci. 2023 Feb 17;24(4):4035. doi: 10.3390/ijms24044035 (PMC9959625; doi:10.3390/ijms24044035)
Supplement: Supplementary file 1 [file ijms-24-04035-s001.zip › ijms-2041318-supplementary/Table_S7.pdf]

Table S7. RAS pathway proteins showing unchanged degree of phosphorylation, but differentially expressed in the proteome

Samples	RAS pathway hyper-expressed proteins with unchanged degree of phosphorylation	RAS pathway hypo-expressed proteins with unchanged degree of phosphorylation
53 (vs yHCs)		
53F (vs aHCs)		PEBP1
53M (vs aHCs)		
67 (vs yHCs)		PEBP1
67F (vs aHCs)		PEBP1
67M (vs aHCs)		GRB2

53 and 67, patients; F, father; M, mother; yHCs, young healthy controls; aHCs, adult healthy controls.
