# Supplementary material for: Changes of RAS Pathway Phosphorylation in Lymphoblastoid Cell Lines from Noonan Syndrome Patients Carrying Hypomorphic Variants in Two NS Genes
Source: Int J Mol Sci. 2023 Feb 17;24(4):4035. doi: 10.3390/ijms24044035 (PMC9959625; doi:10.3390/ijms24044035)
Supplement: Supplementary file 1 [file ijms-24-04035-s001.zip › ijms-2041318-supplementary/Table_S8.pdf]

Table S8. RAS pathway phosphoproteins with impaired expression, not identified in proteome

Samples	RAS pathway hyper-expressed phosphoproteins not identified in proteome	RAS pathway hypo-expressed phosphoproteins not identified in proteome
53 (vs yHCs)	TK1, PAK2	PPP1CA, CASP3, MAPK1
53F (vs aHCs)	PTPN11	PPP1CA, PAK2
53M (vs aHCs)		PPP1CA, MAPK1, PAK2
67 (vs yHCs)	TK1	FNTA, PPP1CA
67F (vs aHCs)	TK1	PPP1CA
67M (vs aHCs)	PAK1	RAC2

53 and 67, patients; F, father; M, mother; yHCs, young healthy controls; aHCs, adult healthy controls.
